# Supplementary material for: Deubiquitinases Sharpen Substrate Discrimination during Membrane Protein Degradation from the ER
Source: Cell. 2013 Aug 1;154(3):609–22. doi: 10.1016/j.cell.2013.06.038 (PMC3732389; doi:10.1016/j.cell.2013.06.038)
Supplement: Document S1. Tables S1 and S2 [file mmc1.pdf]

Table S1. Vpu Constructs Used in This Study, Related to Figures 1–4, 6, and 7
The sequences, modifications, and uses of the Vpu-based constructs in this study. The phosphorylation sites on Vpu are indicated in red in the first construct. TMDs are in cyan. Epitope tags are in green. The TEV protease site is in orange. Oppositely-charged coiled-coil sequences are in magenta. GpA is glycophorin A, VSVG is vesicular stomatitis virus glycoprotein. Epitope tags used are HA (AYPYDVPDYA) and His6 (HHHHHH).

Name	Vector	Sequence Information	Uses in paper
Vpu	pCDNA5/FRT/TO	MVPIIVAIVALVVAIIIAIVVWSIVIIEYRKILRQRKIDRLIDRLIERAEDSGNESEGEVSALVEMGVEMGHHAPWDIDDLENLYFQGAYPYDVPDYA	Fig. 1A, 1B, 2A, 2D, 4D, 4E, S1B, S1C, S1D, S1E, S2A, S2B, S2C, S5C, S5D
Vpu-SN	pCDNA5/FRT/TO	S52N/S56N mutations in cytosolic domain	Fig. 1B, 1C, 2A, 2D, 4D, S1B, S1C, S1D, S1F, S1G, S2A, S5C
Vpu-M1	pCDNA5/FRT/TO	I17F/V21F/V25L mutations in the TMD of Vpu.	Fig. 1A, 4D, S1E, S5C, S5D
Vpu-M2	pCDNA5/FRT/TO	GpA(G13L) TMD in place of Vpu TMD.	Fig. S1C, S1F
Vpu-M3	pCDNA5/FRT/TO	VSVG TMD in place of Vpu TMD.	Fig. S1C, S1F
Vpu-SN-M1	pCDNA5/FRT/TO	Vpu-M1 construct containing SN mutation.	Fig. 1C, S5C
Vpu-SN-M2	pCDNA5/FRT/TO	Vpu-M2 construct containing SN mutation.	Fig. S1C, S1F
Vpu-SN-M3	pCDNA5/FRT/TO	Vpu-M3 construct containing SN mutation.	Fig. S1C, S1F
untagged Vpu	pCDNA3.1	Vpu without any tags	Fig. 7A, 7B, 7C
untagged Vpu-SN	pCDNA3.1	Vpu containing SN mutation without any tags	Used in experiments not shown
untagged Vpu-M1	pCDNA3.1	Vpu-M1 construct without any tags	Fig. 7A, 7C
rec-Vpu	pRSETA	MVPIIVAIVALVVAIIIAIVVWSIVIIEYRKILRQRKIDRLIDRLIERAEDSGNESEGEVSALVEMGVEMGHHAPWDIDDLAYPYDVPDYAGSENLYFQGAHHHHHHST	Fig. 3B, 3C, 3D, 4A, 4B, 4C, 6A, 6C, S3C, S3D, S4B-S4F, S5A, S6A, S7A, S7B, S7C
rec-Vpu-SN	pRSETA	S52N/S56N mutations in cytosolic domain of rec-Vpu	Fig. 3D, S4B
rec-Vpu-M1	pRSETA	I17F/V21F/V25L mutations in TMD of rec-Vpu.	Fig. S4B, S7
rec-Vpu-cyto	pRSETA	MRGSHHHHHHEYRKILRQRKIDRLIDRLIERAEDSGNESEGEVSALVEMGVEMGHHAPWDIDDL	Fig. 3F, S3A
rec-Vpu-SN-cyto	pRSETA	S52N/S56N mutations in rec-Vpu-cyto	Fig. S3A
His-FLAG-Vpu	pRSETA	MRGSHHHHHHGMASMTGGQQMGRDLYDDDDKDRWGSMVPIIVAIVALVVAIIIAIVVWSIVIIEYRKILRQRKIDRLIDRLIERAEDSGNESEGEVSALVEMGVEMGHHAPWDIDDL	Fig. S3B
Coil-Vpu	pET-Duet (with Coil-CD4)	MGSSHHHHHHSQDPGSENLYFQG*LEIRAAFLRQRNTALRTEVAELEQEVQRLENEVSQYETRYGPL*GGGSEYRKILRQRKIDRLIDRLIERAEDSGNESEGEVSALVEMGVEMGHHAPWDIDDLGYPYDVPDYA	Fig. 3F

Table S2. CD4 Constructs Used in This Study, Related to Figures 1–4, 6, and 7
The sequences, modifications, and uses of the CD4-based constructs in this study. The potential ubiquitination sites (lysines) are indicated (red). TMDs are in cyan. Epitope tags are in green. The TEV protease site is in orange. Oppositely-charged coiled-coil sequences are in magenta. GpA is glycophorin A, VSVG is vesicular stomatitis virus glycoprotein. Epitope tags used are FLAG (DYKDDDDK), 3F4 (KTNMKHMAGAAA), and His6 (HHHHHH).

Name	Vector	Sequence Information	Uses in paper
CD4	pCMV	...STPVQPMALIVLGGVAGLLLFIGLGIFFCV RCRHRRRQAERMSQI**K**RLLSE**KK**TCQCPH RFQ**K**TCSPI	Fig. 1A, 1B, 1C, 4D, 7A, 7B, 7C, S1B, S1C, S1D, S1E, S1F, S1G, S5C
CD4-M1	pCMV	GpA TMD in place of the TMD in CD4.	Fig. 1B, 1C, 7A, 7B, 7C, S1B, S1C-S1F, S5C
CD4-M2	pCMV	VSVG TMD in place of the TMD in CD4.	Fig. 1B
CD4-M3	pCMV	Vpu TMD in place of CD4 TMD in CD4.	Fig. S1D, S1G
CD4-Δcyto	pCMV	CD4 lacking the cytosolic domain (ends at R425)	Fig. 1C
CD4	SP64	...STPVQPMALIVLGGVAGLLLFIGLGIFFCV RCRHRRRQAERMSQI**K**RLLSE**KK**TCQCPH RFQ**K**TCSPI	Fig. 1B
mCD4	SP64	MVQPMALIVLGGVAGLLLFIGLGIFFCVRC RHRRRQAERMSQIKRLLSEKKTCQCPHRF QKTCSPIKTNMKHMAGAAA	Fig. 4A, 4B, 4C, 4E, 6A, 6C, S4B, S4C, S4D, S4E, S4F, S5A, S5D, S6A, S7A, S7B, S7C
mCD4-M1	SP64	GpA TMD in place of CD4 TMD in the mCD4.	Fig. 4A, 4B, 4C, 4E, 6A, 6C, S4D, S4E, S5A, S7B
3F4-mCD4	pRSETA	MKTNMKHMAGAAASTPVQPMALIVLGGV AGLLLFI...	Fig. 2C, 2D, S2B, S2C, S3C, S3D
glyco-mCD4	pRSETA	MNKTNMKHMAGAAASTPVQPMALIVLGG VAGLLLFI...	Fig. 2C, 2D
mCD4-4KR	pRSETA	Mutation of cytosolic Lys to Arg in 3F4-mCD4.	Fig. S3C
His-FLAG-mCD4	pRSETA	MRGSHHHHHHGMASMTGGQQMGRDLYD DDDKDRWGSSTPVQPMALIVLGGVAGLLL FI...	Fig. 3C, S3B
rec-mCD4	pRSETA	MSTPVQPMALIVLGGVAGLLLFIGLGIFFC VRCRHRRRQAERMSQIKRLLSEKKTCQCP HRFQKTCSPIDYKDDDDKGSENLYFQGAH HHHHST	Fig. 3B, 3D
rec-CD4-cyto	pRSETA	MMRGSHHHHHHCVRCRHRRRQAERMSQI KRLLSEKKTCQCPHRFQKTCSPIDYKDDDD KGS	Fig. 3F
Coli-CD4	pET-Duet (with Coil-Vpu)	M*GLEIEAAFLERENTALETRVAELRQRVQRLR NRVSQNRTRYGPL*GGGSCVRCRHRRRQAER MSQIKRLLSEKKTCQCPHRFQKTCSPIGDY KDDDDK	Fig. 3F
