# Supplementary material for: Deubiquitinases Sharpen Substrate Discrimination during Membrane Protein Degradation from the ER
Source: Cell. 2013 Aug 1;154(3):609–22. doi: 10.1016/j.cell.2013.06.038 (PMC3732389; doi:10.1016/j.cell.2013.06.038)
Supplement: Document S2. Article plus Supplemental Information [file mmc2.pdf]

Deubiquitinases Sharpen Substrate Discrimination during Membrane Protein Degradation from the ER

Zai-Rong Zhang,[1,3,*] Juan S. Bonifacino,[1] and Ramanujan S. Hegde[2,*]
[1]Cell Biology and Metabolism Program, *Eunice Kennedy Shriver* National Institute of Child Health and Human Development, National Institutes of Health, 18 Library Drive, Bethesda, MD 20892, USA
[2]MRC Laboratory of Molecular Biology, Francis Crick Avenue, Cambridge CB2 0QH, UK
[3]Present address: Nanobiology Institute, Department of Cell Biology, Yale University School of Medicine, Orange, CT 06477, USA
*Correspondence: zairong.zhang@gmail.com (Z.-R.Z.), rhegde@mrc-lmb.cam.ac.uk (R.S.H.)
http://dx.doi.org/10.1016/j.cell.2013.06.038
This is an open-access article distributed under the terms of the Creative Commons Attribution License, which permits unrestricted use, distribution, and reproduction in any medium, provided the original author and source are credited.

SUMMARY

Newly synthesized membrane proteins are queried by ubiquitin ligase complexes and triaged between degradative and nondegradative fates. The mechanisms that convert modest differences in substrate-ligase interactions into decisive outcomes of ubiquitination are not well understood. Here, we reconstitute membrane protein recognition and ubiquitination in liposomes using purified components from a viral-mediated degradation pathway. We find that substrate-ligase interactions in the membrane directly influence processivity of ubiquitin attachment to modulate polyubiquitination. Unexpectedly, differential processivity alone could not explain the differential fates in cultured cells of degraded and nondegraded clients. Both computational and experimental analyses identified continuous deubiquitination as a prerequisite for maximal substrate discrimination. Deubiquitinases reduce polyubiquitin dwell times preferentially on clients that dissociate more rapidly from the ligase. This explains how small differences in substrate-ligase interaction can be amplified into larger differences in net degradation. These results provide a conceptual framework for substrate discrimination during membrane protein quality control.

INTRODUCTION

Eukaryotic integral membrane proteins insert, assemble, and mature at the endoplasmic reticulum (ER) (Skach, 2009). Membrane proteins that fail to mature are ubiquitinated by ER resident ubiquitin ligases and degraded by the proteasome in a process termed ER-associated degradation (ERAD) (Vembar and Brodsky, 2008). ERAD pathways are also utilized for regulatory control of membrane proteins on the basis of cellular demand (Hampton, 2002; DeBose-Boyd, 2008). Thus, all newly synthesized membrane proteins are triaged between degradative and nondegradative fates at the ER.

Proper triage is critical to cellular homeostasis and physiology. Failure to efficiently target misfolded proteins for degradation can lead to proteotoxicity, cell death, and disease (Chiti and Dobson, 2006). Conversely, overaggressive surveillance would result in degradation of functional products and loss-of-function consequences (Grove et al., 2011). Inappropriate triage of regulated factors can also lead to imbalanced expression levels with pathologic effects (DeBose-Boyd, 2008). Thus, achieving the correct balance in membrane protein triage is vital, but the mechanisms that regulate client discrimination are not well understood.

A key step in triage between degradative and nondegradative fates is client ubiquitination by an ER-resident ubiquitin ligase (Hirsch et al., 2009). A polyubiquitin tag serves as a signal for downstream factors to extract the membrane protein and deliver it to the proteasome for degradation (Vembar and Brodsky, 2008). The acquisition or not of polyubiquitin is therefore a deciding factor for client triage. The simplest model is one where clients destined for degradation are recruited to the ligase, whereas other proteins are not. This paradigm of discrimination, based solely on ligase access, is common in regulated degradation. For example, Sic1 is targeted for degradation only after phosphorylation generates a mark for ubiquitin ligase recruitment (Feldman et al., 1997). Although attractively simple, there are several reasons to suspect that ligase access alone cannot provide sufficient discriminatory power in quality control.

First, quality control is usually thought to involve broad "surveillance" of many potential clients, with commitment for degradation of only a minor subset. Second, most quality-control ligases must recognize a highly diverse range of clients; not only are there numerous individual proteins, but each one can be misfolded in myriad ways. This means that a single "recognition motif" or degron is unlikely, placing constraints on the level of selectivity that can be achieved by simple interaction. And third, essentially all newly synthesized proteins are potential clients early in their biosynthesis before they have folded, assembled, and matured. Thus, the issue may be less about

whether a client interacts with a ligase, but rather more subtle aspects of the nature of the interaction.

Indeed, coimmunoprecipitation (co-IP) and crosslinking studies show qualitatively similar ligase interactions for degraded and nondegraded versions of potential clients. For example, the client Hmg2p interacts with the ligase Hrd1p regardless of whether Hmg2p is subsequently degraded or not (Gardner et al., 2001; Sato et al., 2009). Similarly, the ligase-client interactions for degraded and nondegraded forms of TCRα are also remarkably similar (Ishikura et al., 2010). Thus, ligase access per se does not obligate client degradation; rather, a more nuanced model is needed to explain how modest differences in ligase interaction are converted to large differences in client fate.

Efforts to study this process in mechanistic depth have been hampered by various challenges. In vitro systems to date are typically composed of microsomes (or semipermeabilized cells) combined with cytosol (Shamu et al., 1999; Nakatsukasa et al., 2008; Garza et al., 2009). Although the cytosol is experimentally accessible, the membrane components remain a challenge. Full control of reactions in the membrane requires the capacity to independently reconstitute purified client and ligase complexes. Unfortunately, a misfolded protein client poses considerable obstacles to expression, purification, and reconstitution. Furthermore, the multiple components and complex topology of ER-resident ligases (Hirsch et al., 2009) has thus far precluded their functional membrane reconstitution. Thus, it has not been possible to rigorously examine how the nature of client-ligase interactions in the membrane influences ubiquitination and commitment for degradation.

To circumvent these issues, we turned to virus-mediated degradation of host membrane protein from the ER. Several viruses encode membrane proteins that exploit host ubiquitin ligases for regulated degradation of key cellular proteins (Isaacson and Ploegh, 2009). Such systems have yielded key insights into ERAD previously and provide several attractive features for reconstitution studies. First, the client is not recognized on the basis of misfolding, meaning that a native protein amenable to recombinant production can be used. Second, the system is typically orthogonal to endogenous pathways and utilizes a single defined ligase. Third, viral systems are usually rapid, efficient, and robust. Thus, viral systems of regulated degradation often encapsulate the salient features of a physiologic process in a simplified format.

In this study, we exploit these advantages to reconstitute with purified components the ubiquitination of CD4 by the HIV-encoded protein Vpu. The reconstituted system permitted quantitative mechanistic analysis to reveal key aspects of substrate discrimination and commitment for degradation. We discovered that modest differences in client-ligase interaction are converted into clear differences in polyubiquitination by a combination of processivity differences and deubiquitinases (DUBs) that impact the dwell time of a degradation mark on potential clients. These findings have implications for the mechanism underlying membrane protein triage and quality control at the ER.

RESULTS AND DISCUSSION

Experimental System

The HIV-encoded membrane protein Vpu interacts with CD4 at the ER and routes it for proteasomal degradation (reviewed by Nomaguchi et al., 2008; Figure S1A available online). Although Vpu is not a ubiquitin ligase, its phosphorylation at two sites by casein kinase 2 (CK2) permits a high-affinity interaction with the SCF$^{\beta TrCP}$ ubiquitin ligase complex via its F-box protein βTrCP (Margottin et al., 1998). Ligase recruitment to the ER leads to ubiquitination of the unstructured cytosolic tail of CD4. Phospho-Vpu therefore converts the nucleocytoplasmic SCF$^{\beta TrCP}$ complex into an ER ubiquitin ligase that mediates selective degradation of its CD4 client. Importantly, the basic features of this system work in various heterologous systems (Chen et al., 1993; Schubert et al., 1998; Meusser and Sommer, 2004), arguing against cell-type-specific features in CD4 degradation. The Vpu-mediated CD4 degradation pathway therefore affords a simplified model of membrane protein degradation whose mechanistic details may be amenable to dissection by in vitro reconstitution. Our strategy was to first define versions of CD4 and Vpu that differ in their membrane interactions and degradation and then use these model clients and nonclients to investigate the basis of substrate discrimination during membrane protein triage.

Transmembrane-Domain-Dependent Interactions Modulate CD4 Degradation by Vpu

CD4 expression in cultured cells was downregulated in a dose-dependent manner by coexpression of Vpu (Figure 1A). A phosphorylation site Vpu mutant (Vpu-SN; see Table S1 for construct details) was completely inactive (Figure S1B), as characterized in earlier work (Schubert and Strebel, 1994; Magadán et al., 2010). Changing three residues in the transmembrane domain (TMD) of Vpu (I17F/V21F/V25L, termed Vpu-M1) largely abolished its ability to downregulate CD4 (Figure 1A). Similar results were seen when the Vpu TMD was replaced with heterologous TMDs (Vpu-M2, and Vpu-M3; Figure S1C). Replacing the TMD of CD4 with heterologous TMDs (CD4-M1 and CD4-M2) also rendered it refractory to Vpu downregulation (Figure 1B) unless the heterologous TMD (CD4-M3) could interact with Vpu (Figure S1D). Importantly, the various CD4 and Vpu TMD mutant proteins were verified by fractionation experiments to be membrane inserted (data not shown). Results from the steady-state experiments with the M1 mutants were confirmed by pulse-chase analysis (Figure S1E). Thus, Phospho-Vpu-mediated CD4 degradation from the ER depends on features of the TMDs of both proteins (Magadán and Bonifacino, 2012).

To examine the contribution of TMDs to the CD4-Vpu interaction, we performed co-IP experiments. The Vpu-SN phosphorylation mutant permitted interactions to be assessed in the absence of downstream degradation. As expected (Bour et al., 1995; Magadán et al., 2010), Vpu-SN coimmunoprecipitated CD4 (Figure 1C) and vice versa (Figure S1F). However, a qualitatively similar interaction by co-IP was also observed with TMD mutants of either Vpu or CD4 (Figures 1C, S1F, and S1G). Deleting the cytosolic domain of CD4 showed a marked (>80%), but not complete, loss in co-IP with Vpu (Figure 1C), consistent with an interaction between their cytosolic tails (Bour et al., 1995). Combining the CD4 cytosolic deletion with a Vpu TMD mutant abolished the interaction entirely (data not shown). Thus, the CD4-Vpu interaction is bipartite via both the cytosolic and TMD regions.

The contribution of the TMD was judged to be relatively minor based on the modest ~30% reduction in co-IP with the CD4 or

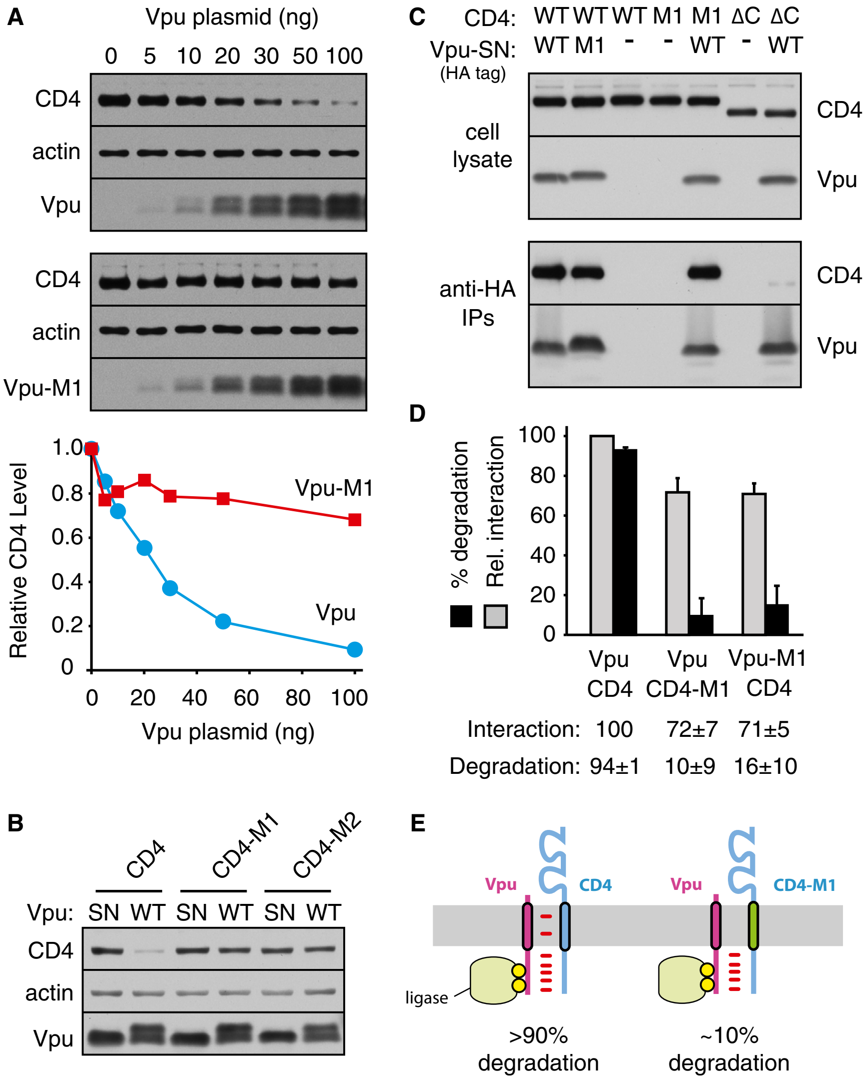

Figure 1. TMD-Dependent CD4 Degradation by Vpu

(A) Increasing amounts of Vpu (top) or Vpu-M1 (panel) plasmids were cotransfected with a fixed amount (100 ng) of CD4 and analyzed 24 hr later by immunoblotting. The two Vpu bands correspond to phosphorylated and unphosphorylated forms. Actin is a loading control. Quantification of CD4 is shown below the blots.

(B) Wild-type (WT) Vpu or the Vpu-SN phosphorylation mutant (SN) was coexpressed with the indicated CD4 constructs and analyzed by immunoblotting.

(C) HA-tagged Vpu-SN constructs containing the wild-type or M1 mutant transmembrane domain were coexpressed with the indicated CD4 constructs and analyzed by immunoblotting directly (5% lysate; top) or after IP with anti-HA antibodies (bottom).

(D) Quantification of CD4 degradation and interaction (by co-IP) for the indicated Vpu-CD4 pairs. Degradation was analyzed as in (B), and interaction was analyzed as in (C). Mean ± SD from at least three experiments.

(E) Disrupting TMD-TMD interactions between Vpu and CD4 markedly reduces CD4 degradation despite minimal effects on their overall interaction. See also Figure S1 and Tables S1 and S2.

Vpu M1 mutants (Figure 1D). This indicates that the TMD mutant proteins are engaged in CD4-Vpu complexes to at least 70% the level as the wild-type pair. Yet, phospho-Vpu-dependent CD4 degradation is almost completely lost (quantified in Figure 1D), suggesting that even small decreases in interaction are sufficient to effect a large change in degradation. This mirrors earlier observations with ER ubiquitin ligases, where degraded versus nondegraded versions of clients displayed surprisingly minor differences in ligase interaction (Gardner et al., 2001; Meacham et al., 2001; Sato et al., 2009; Ishikura et al., 2010). Thus, the Vpu-CD4 system and their TMD mutants provide a simple model system to dissect the mechanistic basis of selective degradation for subtly different client-ligase interactions in the membrane (Figure 1E).

In Vitro Reconstitution of CD4 Ubiquitination by a Vpu-Bound Ubiquitin Ligase

To facilitate in vitro reconstitution of Vpu-mediated CD4 ubiquitination, we isolated ER microsomes from cells expressing HA-tagged Vpu or phosphorylation-deficient Vpu-SN. Coimmunoprecipitation experiments showed that Vpu, but not Vpu-SN, was associated with the $SCF^{\beta TrCP}$ complex (Figure 2A). The Vpu-$SCF^{\beta TrCP}$ complex was completely stable to 0.8 M salt wash (Figure S2A), illustrating that this complex essentially behaves as an integral membrane ubiquitin ligase. Importantly, the microsomes were functional for protein translocation (Figure 2B), allowing ^{35}S-labeled proteins to be introduced into them by in vitro translation.

Given that Vpu has at most one or two residues in the ER lumen, we reasoned that the only relevant portion of CD4 for its selective ubiquitination was the TMD and cytosolic tail. A "mini-CD4" (mCD4) containing only these domains was constructed (Table S2) and verified to insert efficiently and correctly into ER microsomes by in vitro translation (Figure 2C). Although this construct does not contain a signal peptide, the TMD, now being positioned near the N terminus, acts as a type I signal anchor to mediate targeting and insertion (Higy et al., 2004). Protease protection assays combined with IPs verified that mCD4 acquires the correct type I topology (Figure 2C). Appending an N-terminal glycosylation site to mCD4 resulted in mCD4 glycosylation (Figure 2C), corroborating its insertion by an independent means.

Following mCD4 in vitro translation and insertion, microsomes were isolated and washed with 0.8 M salt to remove the cytosol and any noninserted mCD4. Next, ubiquitination reactions were

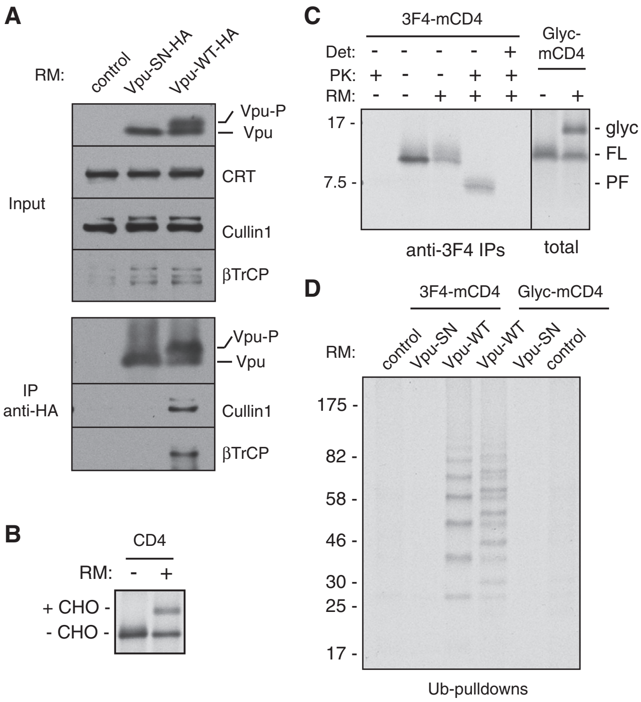

Figure 2. Reconstitution of Vpu-Mediated CD4 Ubiquitination on Microsomes

(A) HEK293-derived rough microsomes (RM) from cells expressing nothing (control), Vpu-HA, or Vpu-SN-HA were analyzed directly or after anti-HA IPs. The positions of phosphorylated and unphosphorylated Vpu are indicated. CRT is the ER resident chaperone calreticulin.

(B) CD4 in vitro translated in the presence of HEK293 RMs is translocated as evidenced by its glycosylation.

(C) 3F4-mCD4 was in vitro translated with ^{35}S-methionine in the absence or presence of RMs and subjected to digestion with proteinase K (PK) in the absence or presence of detergent (Det). A matched construct containing a consensus glycosylation site (Glyc-mCD4) was also analyzed. The products were immunoprecipitated using antibodies against the N-terminal 3F4 tag and analyzed by SDS-PAGE and autoradiography. The bands are as follows: FL, full-length mCD4; glyc, glycosylated mCD4; PF, protease-protected fragment.

(D) 3F4-mCD4 or Glyc-mCD4 was in vitro translated and translocated into the indicated RMs from HEK293 cells, and the isolated, high-salt washed RMs were subjected to a ubiquitination reaction by adding E1 and E2 enzymes, His-tagged ubiquitin, and ATP. Ubiquitinated products were pulled down via the His tag and the mCD4 (or Glyc-mCD4) visualized by autoradiography.

See also Figure S2 and Tables S1 and S2.

initiated by adding purified E1 and E2 enzymes, together with tagged-ubiquitin and ATP. Ubiquitin pull-downs followed by visualization of the radiolabeled mCD4 revealed a ladder of ubiquitinated mCD4 (Figure 2D). Glycosylated mCD4 was also ubiquitinated (Figure 2D), verifying that membrane-inserted mCD4 was being modified. Ubiquitination was dependent on SCF$^{\beta TrCP}$ because a parallel reaction with phosphorylation-deficient Vpu-SN failed to be ubiquitinated (Figure 2D), consistent with its inability to coimmunoprecipitate SCF$^{\beta TrCP}$ (Figures 2A and S2A). Furthermore, the absolute dependence on phospho-Vpu illustrates that endogenous ligases in this system do not perceive mCD4 as a misfolded protein for ubiquitination. Optimal polyubiquitination was achieved using a mixture of UbcH3 and UbcH5 (Figure S2B), consistent with different E2 enzymes being optimal for initial ubiquitination versus chain elongation (Rodrigo-Brenni and Morgan, 2007; Wu et al., 2010). Use of a K48R mutant ubiquitin showed deficient chain elongation (Figure S2C). Ubiquitination was not observed when the four lysines in the tail of CD4 were mutated to arginines (data not shown). Thus, the Vpu-SCF$^{\beta TrCP}$ ligase complex ubiquitinates mCD4 on isolated membranes in vitro, setting the stage for reconstitution with purified factors.

Reconstitution of Membrane Protein Ubiquitination with Recombinant Components

The modularity of the Vpu-CD4 system afforded the opportunity to assemble it from individual recombinant factors in liposomes (Figure 3A). We expressed and purified mCD4 and Vpu from *E. coli* (Figure 3B), confirmed that purified Vpu can be phosphorylated efficiently by purified Casein Kinase 2 (CK2; data not shown), and that the cytosolic domain interacted efficiently with βTrCP in a phosphorylation-dependent manner (Figure S3A). We then optimized conditions for reconstitution of mCD4 and Vpu into synthetic liposomes such that most of it was incorporated in the correct orientation as determined by protease protection assays (Figure S3B). Co-IP experiments showed that when mCD4 and Vpu were coincorporated into the same liposomes, they interacted with each other (Figure 3C). By contrast, mixing separate liposomes each containing mCD4 or Vpu, followed by solubilization and IP, showed minimal interaction (Figure 3C).

Incubation of liposomes containing mCD4 and Vpu with purified recombinant SCF$^{\beta TrCP}$ complex (Figure 3B), E1 and E2 enzymes, ubiquitin, and ATP resulted in polyubiquitination of mCD4 (Figure 3D). Ubiquitination was strictly dependent on Vpu phosphorylation because neither unphosphorylated wild-type Vpu nor CK2-treated Vpu-SN supported mCD4 ubiquitination (Figure 3D). As expected from earlier work (Duda et al., 2008; Saha and Deshaies, 2008), the speed and efficiency of ubiquitination was improved by Nedd8 modification of the SCF$^{\beta TrCP}$ complex (Figures S3C and S3D).

The cytosolic domains of CD4 and phospho-Vpu, despite the capability to interact weakly (Singh et al., 2012), failed to produce substantial CD4 ubiquitination (Figures 3E and 3F). By contrast, tethering these His-tagged cytosolic domains to the surface of membranes via Ni^{2+}-NTA lipid permitted ubiquitination (Figures 3E and 3F). However, the ubiquitination was substantially less processive than membrane-inserted mCD4 and Vpu as evidenced by fewer ubiquitins on the substrate (around three to seven, compared to over ten). This effect was not due to membrane insertion per se because replacing the TMDs of Vpu and mCD4 with oppositely charged interacting coiled coils permitted highly processive ubiquitination in the absence of any membrane (Figures 3E and 3F).

These results rigorously illustrate that Vpu-mediated CD4 ubiquitination requires no additional factors beyond the SCF$^{\beta TrCP}$ complex, its associated E2 enzymes, E1, and ubiquitin. Moreover, the data suggest that the cytosolic domains of CD4 and Vpu interact too weakly to mediate efficient ubiquitination in solution. However, constraining them to a membrane surface allows some ubiquitination, presumably by limiting their diffusion

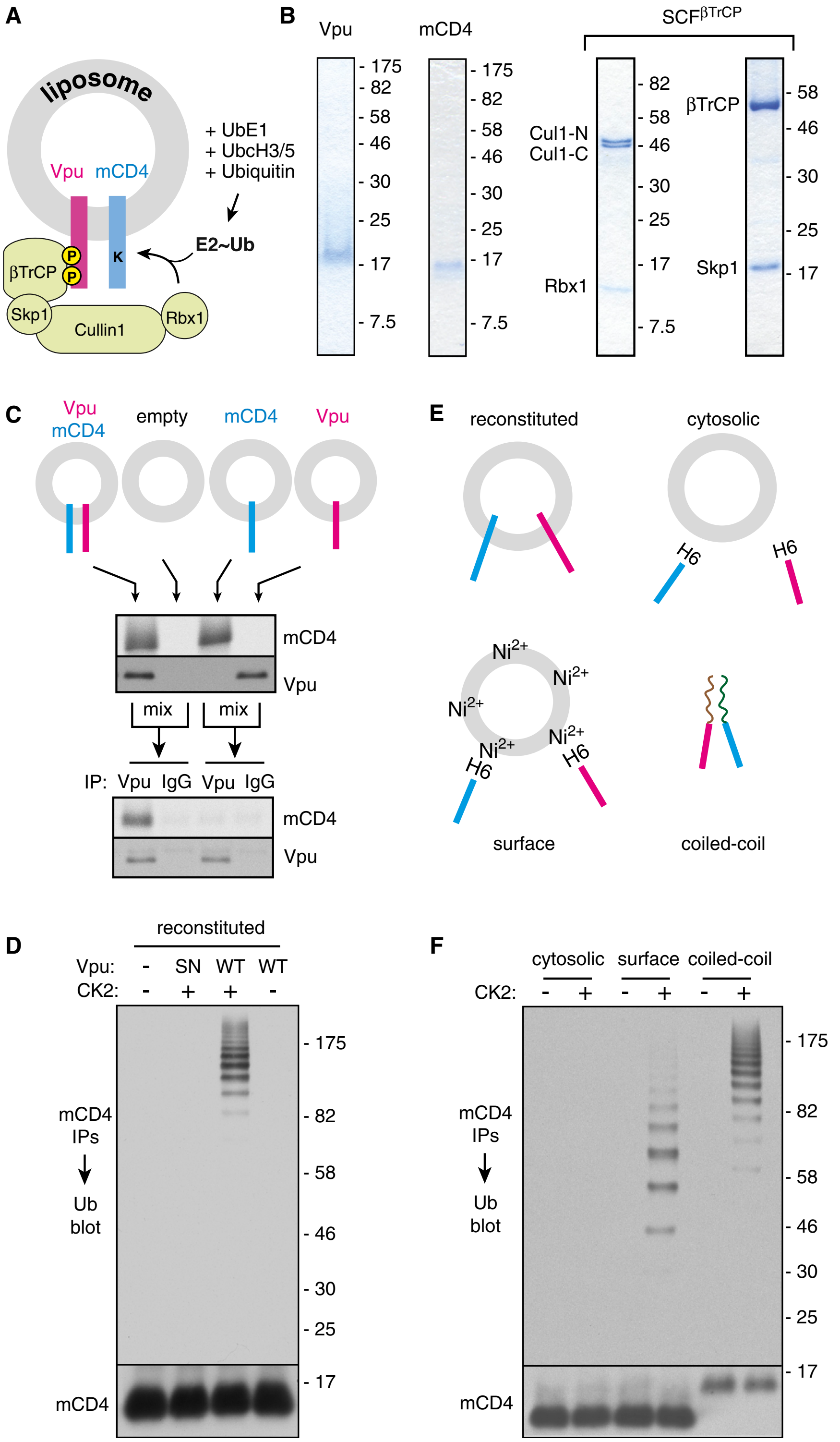

Figure 3. TMD-Dependent mCD4 Ubiquitination with Purified Factors

(A) Diagram depicting the purified system.

(B) Coomassie-stained gels of recombinant purified proteins.

(C) Co-IP analysis of recombinant Vpu and mCD4 in reconstituted proteoliposomes. Radiolabeled mCD4 and recombinant Vpu were incorporated either separately or together into liposomes, and the indicated samples were mixed, solubilized, and subjected to IP with anti-Vpu or control IgG. The samples before and after IP were analyzed by immunoblotting for Vpu and autoradiography for mCD4, respectively.

(D) Liposomes reconstituted with mCD4 alone, with Vpu (WT), or with Vpu-SN were incubated with CK2 (as indicated) and ubiquitination factors (E1, UbcH3, UbcH5c, Myc-ubiquitin, ATP). mCD4 was immunoprecipitated and the samples blotted for ubiquitin or mCD4.

(E and F) Vpu and mCD4 combinations as depicted in (E) were subjected to ubiquitination and analyzed as in (D).

See also Figure S3 and Tables S1 and S2.

and degrees of freedom to enhance their interaction. The TMDs add to this interaction, presumably by orienting the cytosolic domains and by interacting within the membrane to further stabilize the Vpu-CD4 complex. Prolonged interaction permits the ligase time to sequentially build long ubiquitin chains. This establishes a membrane protein ubiquitination reaction that relies on TMD-dependent substrate-ligase interaction to maximize processivity.

Substrate-Ligase Interactions Modulate Ubiquitination Processivity

We next asked whether the discrimination of correct from incorrect substrates observed in cultured cells could be recapitulated in vitro. To facilitate quantitative analysis, we modified the recombinant system to contain radiolabeled substrate generated by in vitro translation (Figure S4A). The translated and microsome-inserted substrate was separated from the translation extract and bulk microsomal proteins and mixed with purified lipids and recombinant Vpu prior to reconstitution into proteoliposomes. The final proteoliposomes in this radiolabeled recombinant system contained Vpu and radiolabeled mCD4, but undetectable levels of proteins from the original in vitro translation reaction and more than 95% removal of microsomal proteins (data not shown). The remainder of the components (for ubiquitination) was from recombinant sources. As expected, mCD4 ubiquitination in the radiolabeled recombinant system displayed a strict dependence on phospho-Vpu, showed high processivity and was easily quantifiable

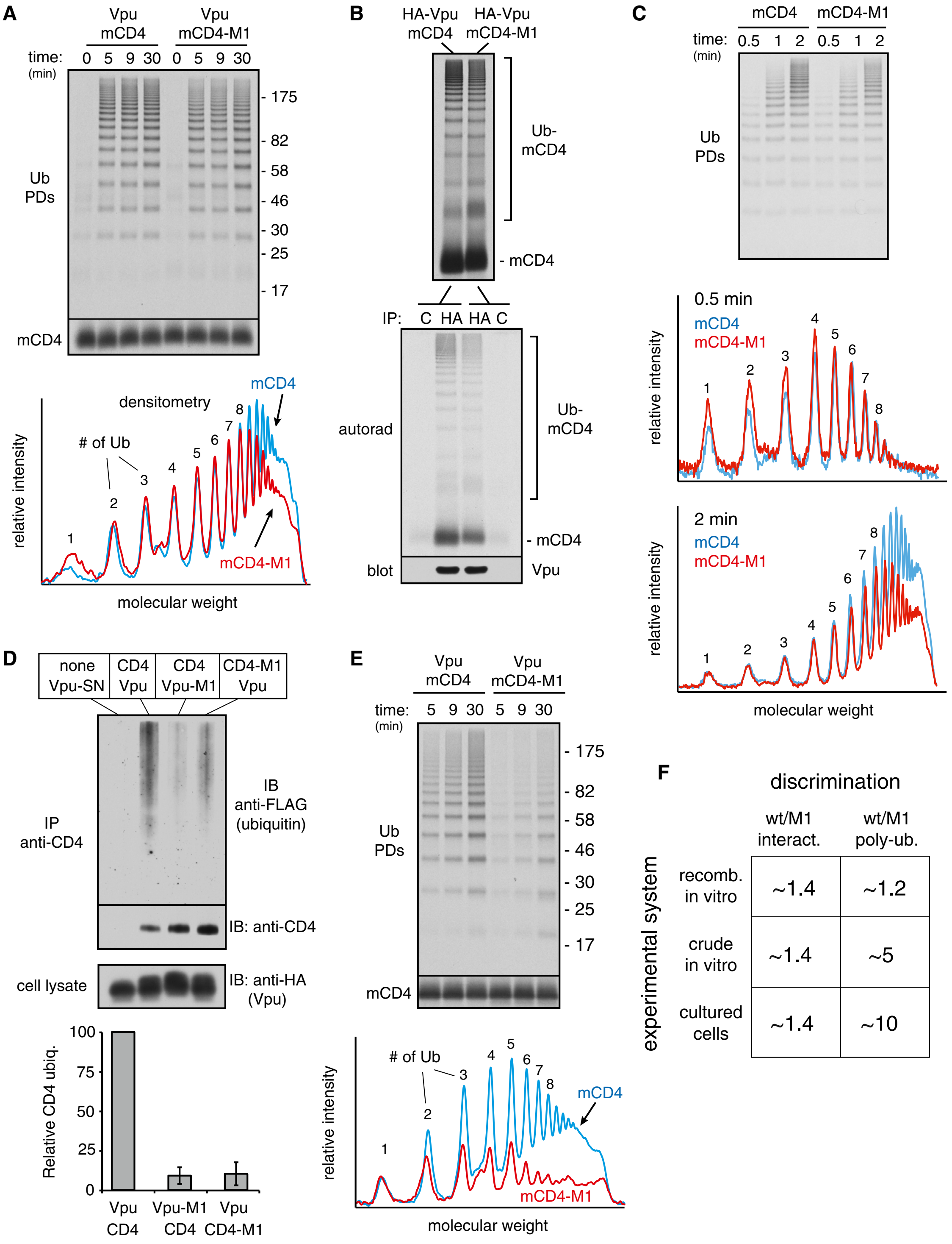

Figure 4. Substrate Discrimination in Cells, In Vitro, and in Purified Systems

(A) Analysis of discrimination in a radiolabeled recombinant ubiquitination system. Radiolabeled mCD4 (or mCD4-M1) isolated from in vitro translation reactions (see Figure S4A) was coreconstituted with recombinant Vpu into liposomes. The purified proteoliposomes were subjected to ubiquitination with recombinant SCF$^{\beta TrCP}$, purified E1 and E2 enzymes, His6-tagged ubiquitin, and ATP. The reaction at different time points was stopped and analyzed for ubiquitinated mCD4 via pull-downs of the tagged ubiquitin (Ub PDs). Total mCD4 in the reaction is shown in the bottom panel. The graph below the autoradiograph depicts the densitometry profiles of the 5 min samples, with the individual ubiquitinated species indicated.

(B) Ubiquitination reactions as in (A) were either analyzed directly (top) or after solubilization and native IP using anti-HA or control antibodies (bottom). The HA-tagged Vpu recovered in the IP was analyzed by blotting, and the mCD4 was analyzed by autoradiography.

(legend continued on next page)

given that only the substrate was radiolabeled (Figures S4B and S4C).

Unexpectedly, the Vpu-mediated ubiquitination of mCD4 and mCD4-M1 was nearly identical (Figure 4A). Co-IP experiments showed that the Vpu-mCD4-M1 interaction in vitro was reduced modestly relative to the Vpu-mCD4 interaction (Figure 4B), very similar to co-IP experiments in cells (Figure 1D). Furthermore, crosslinking analysis in proteoliposomes showed that βTrCP crosslinks almost equally well to mCD4 versus mCD4-M1 (Figure S4D). Importantly, ubiquitination was strictly dependent on the Vpu-SCF complex (Figures S4B and S4E), arguing against any other contaminating activities in the system that could explain the lack of difference. Thus, discrimination of correct from incorrect clients in the minimal in vitro system was incongruent with degradation efficiencies observed in cultured cells.

Similar results were observed when Vpu-M1 was used for mCD4 ubiquitination (Figures S4C and S4F). The lack of discrimination was observed over a wide range of concentrations (Figure S5A) and could not be easily explained by excessively high (or low) substrate or ligase levels. However, careful quantification of the ubiquitination profiles from the in vitro reactions revealed a subtle but highly reproducible difference in the high molecular weight species (see densitometry profiles, Figures 4A, S4F, and S5B). This reduction reflects a slight deficiency in building particularly long chains for the mutant mCD4-Vpu pairs relative to the wild-type pair, suggesting reduced processivity of ubiquitin addition.

Indeed, analysis of ubiquitination profiles with finer temporal resolution showed that at very short time points (30 s), mCD4-M1 was indistinguishable from mCD4 (Figure 4C). Only at later times is a difference seen preferentially for longer ubiquitin chain lengths. Thus, ubiquitins are initially added identically to mCD4 and mCD4-M1, consistent with the crosslinking results showing that Vpu-bound βTrCP "sees" these two proteins almost equally well (Figure S4D). However, mCD4-M1 presumably dissociates slightly faster from the Vpu-ligase complex than mCD4. This would give mCD4-M1 slightly less time than mCD4 to acquire ubiquitins. Although this may not be significant for a single encounter, the additive effect of multiple encounters would explain the difference preferentially seen for long ubiquitin chains. We therefore conclude that modest differences in interaction translate to differences in processivity of ubiquitin addition that translate to differences in long-chain modified substrates.

Discrepant Substrate Discrimination in Reconstituted versus Native Systems

Although the processivity differences in ubiquitination in vitro are consistent with similarly subtle differences in interaction by co-IP, neither result matches the substantially greater difference in degradation for wild-type versus mutant TMDs. Indeed, Vpu-mediated CD4 ubiquitination in cultured cells under conditions of proteasome inhibition showed a clear ~5- to 10-fold difference between CD4 and CD4-M1 (Figure 4D). This corresponded not only to decreased degradation (e.g., Figure 1), but also to decreased dislocation as judged by the absence of a deglycosylated product upon proteasome inhibition (Figure S5C). The cultured cell system therefore converts modest differences in CD4-Vpu interaction into substantial differences in CD4 polyubiquitination, dislocation, and degradation. This feature of discrimination is only partially recapitulated in the radiolabeled recombinant system as a slight difference in processivity (Figure 4A). By contrast, use of ER microsomes instead of reconstituted proteoliposomes displayed clear discrimination between wild-type and mutant mCD4-Vpu pairs (Figures 4E and S5D). Thus, the radiolabeled recombinant in vitro system seems to lack some feature(s) that imparts substrate discrimination to the crude in vitro and cellular systems (summarized in Figure 4F).

Kinetic Modeling Suggests a Role for Deubiquitination in Substrate Discrimination

A clue to the missing feature came from the observation that enhanced discrimination in the crude in vitro system was accompanied by shorter ubiquitin chains (Figure 5A). This suggested that the crude system contained deubiquitination activity, which has been speculated to influence the timing of degradation from the ER (Blount et al., 2012; Lederkremer and Glickman, 2005; Feldman and van der Goot, 2009). Indeed, ubiquitinated mCD4 produced in the radiolabeled recombinant system was deubiquitinated when incubated in a crude cell lysate (Figure S6A). Ubiquitin removal occurred progressively from the distal end of the chain (Figure S6B), illustrating that mCD4 is subject to exodeubiquitination.

To understand how deubiquitination can influence substrate discrimination, we turned to kinetic modeling. Pioneering studies by Pierce et al. (2009) established a kinetic description of $SCF^{\beta TrCP}$-mediated ubiquitination of β-catenin peptide. We expanded and revised the kinetic model in a few minor ways (Figures 5B and S6C) and verified that it produced a ubiquitination profile roughly comparable to that observed experimentally (Figure 5C, green bars, compare to Figure 5A).

We then incorporated DUBs into the model such that they can remove the terminal ubiquitin from the chain. Using a DUB concentration of 0.4 μM, and a K_{cat} of 0.5 s^{-1} (Hassiepen et al., 2007), the model produces a ubiquitination profile similar to that observed experimentally in the crude in vitro Vpu-mCD4 system (Figure 5C, orange bars, compare to Figure 5A). Using

(C) Analysis of ubiquitination reactions as in (A) at short time points.
(D) CD4 ubiquitination in cells. The indicated combinations of Vpu and CD4 were cotransfected with FLAG-ubiquitin, treated with proteasome inhibitor (40 μM MG132) for 5 hr, subjected to IP with anti-CD4, and analyzed by immunoblotting for FLAG-ubiquitin and CD4. Vpu levels in the lysate are also shown. The graph shows quantification (mean ± SD; n = 3) of relative CD4 ubiquitination normalized to total CD4.
(E) Ubiquitination reactions of in vitro translated mCD4 or mCD4-M1 in Vpu-containing HEK293 microsomes. Quantification of the 30 min time point is shown below the autoradiograph.
(F) Summary of the results of interaction and ubiquitination analysis from cell culture, crude in vitro, and recombinant in vitro systems. The approximate ratios of wild-type (wt) to M1 mutant recovered by co-IP (interaction) and observed to be polyubiquitinated are indicated. The recombinant in vitro system shows poor discrimination.
See also Figures S4 and S5 and Tables S1 and S2.

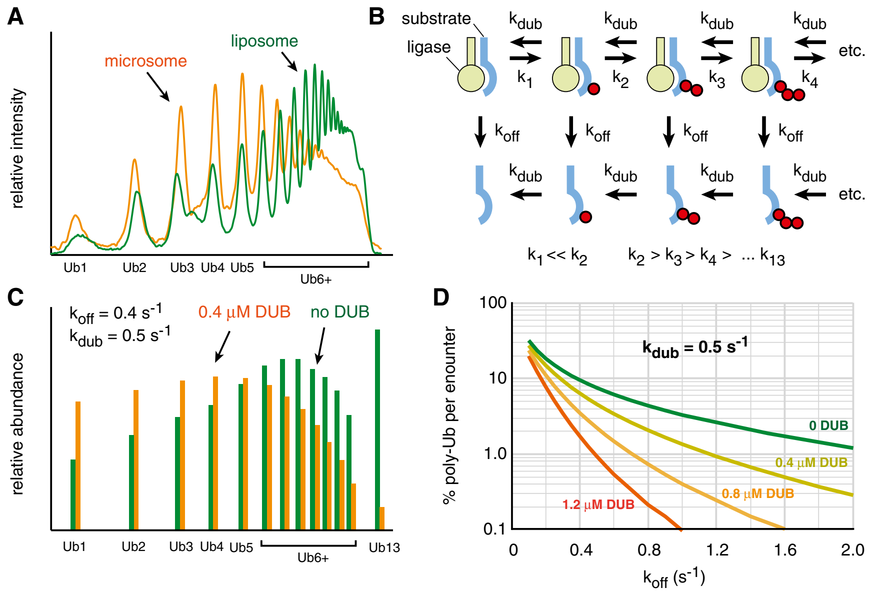

Figure 5. Kinetic Modeling of Ubiquitination without DUBs versus with DUBs

(A) Normalized ubiquitination profiles of reactions performed in liposomes (green) versus microsomes (orange).

(B) Kinetic model of substrate ubiquitination, substrate dissociation, and deubiquitination. See Figure S6C for additional details.

(C) Output of the kinetic model at 20 s using the indicated parameters without (green) or with (orange) DUBs. The relative amounts of the individual ubiquitinated species are plotted.

(D) Relationship between polyubiquitination (defined as four or more ubiquitins), k_{off}, and DUB activity predicted by the model analyzed at 20 s.

See also Figure S6.

this as a starting point, we used the model to examine the role of DUB activity in the relationship between client-ligase interaction and polyubiquitination. In the absence of DUB activity, we observed that substrate polyubiquitination (defined here as four or more ubiquitins) in a single encounter decreased progressively with increasing K_{off} values for the substrate-ligase interaction (Figure 5D). This is easily rationalized because rapid substrate dissociation from the ligase provides less time to build ubiquitin chains. In the presence of DUB activity, a qualitatively similar relationship between K_{off} and polyubiquitination was observed (Figure 5D). However, overall polyubiquitination was less than without DUBs (particularly at higher K_{off} values), and the curve was progressively steeper with increasing DUB concentration.

The net effect of this is to increase discrimination (as defined by fold differences in polyubiquitination) between two substrate-ligase pairs of differing rates of dissociation (K_{off}). For example, in the absence of DUBs (Figure 5D, green curve), a 2-fold increase in K_{off} (from 0.4 to 0.8) results in an ~2.2-fold decrease in polyubiquitination (from 9.4% to 4.3%). In the presence of high DUB activity (Figure 5D, red curve), that same 2-fold increase in K_{off} results in an ~8.3-fold decrease in polyubiquitination (from 1.66% to 0.2%). Thus, DUB activity can noticeably improve substrate discrimination in this model, albeit at the expense of overall ubiquitination efficiency. This point is also revealed by inspecting the kinetic model output at different time points (Figures S6D and S6E).

DUB Activity Enhances Substrate Discrimination by Vpu

Adding cytosol to the radiolabeled recombinant system during the ubiquitination reaction decreased overall polyubiquitination and ubiquitin chain length and increased discrimination between mCD4 and mCD4-M1 (Figure 6A). Pretreatment of the cytosol with Ubiquitin-aldehyde partially reversed each of these effects, illustrating that their origin was likely to involve DUBs (Figures 6A and 6B). The same results, albeit somewhat muted, were observed when the mCD4-Vpu-M1 pair was analyzed (Figure S7A). The incomplete reversal with ubiquitin-aldehyde can be explained by incomplete inhibition of DUB activity (Figure S7B), whereas the slightly reduced discrimination over time seems to be due to DUBs losing activity sooner than the SCF (Figure S7C). These technical quirks notwithstanding, at least one contributing factor to discrimination imparted by cytosol can be attributed to DUB activity.

Use of a purified DUB (the catalytic domain of USP2) in lieu of cytosol also imparted discrimination to the radiolabeled recombinant mCD4-Vpu system in a concentration-dependent manner (Figures 6C–6E). Consistent with predictions from the kinetic modeling, discrimination came at the expense of ubiquitination efficiency (Figure 6F). Other purified DUBs (USP7, Ataxin3) could also impart discrimination in this assay, albeit to different extents that correlated with their relative catalytic activities (data not shown). It therefore appears that DUB activity, rather than a specific DUB, is the crucial determinant in imparting discrimination in this system.

Evidence for a Role of DUBs in Substrate Discrimination in Cultured Cells

An important insight from our in vitro and modeling studies is that a nondegraded client (i.e., CD4-M1) is nevertheless ubiquitinated, but that the ubiquitin chains are rapidly counteracted and rarely long enough to trigger degradation. Thus, lack of degradation is not due to a lack of ligase interaction or ubiquitination, but to rapid deubiquitination. This conclusion makes two testable predictions. First, nondegraded clients should be transiently modified with ubiquitin chains that do not usually reach a sufficient length to trigger degradation. Second, reducing cellular DUB activity should mute substrate discrimination by preferentially reducing the levels of normally nondegraded substrates. Each of these predictions was examined to evaluate the role of DUBs in cultured cells.

We analyzed ubiquitination of clients without proteasome inhibitor pretreatment and attempted to detect individual ubiquitinated species rather than the smear typically seen with prolonged inhibitor-treated samples. Strikingly, ubiquitinated

products were observed at similar levels for CD4 in the presence of Vpu, CD4-M1 in the presence of Vpu, and CD4 in the presence of Vpu-M1 (Figure 7A). However, the ubiquitin chains were clearly different: whereas CD4 intended for degradation contained a substantial amount of polyubiquitin, CD4-M1 contained primarily one to three ubiquitins. This difference is readily apparent when the ubiquitination profiles are plotted and normalized to the amount of substrate (Figure 7A, graph). The same was observed for CD4 coexpressed with Vpu-M1.

With proteasome inhibitor pretreatment for 3 hr, polyubiquitination of CD4 was increased, consistent with its eventual destiny of degradation (Figure 7B). By contrast, the oligoubiquitination of CD4-M1 decreased modestly (Figure 7B). With even longer proteasome inhibition, the oligoubiquitin species become undetectable relative to the polyubiquitin smear, which preferentially accumulates on CD4 and not CD4-M1 (e.g., see Figure 4D). This can be explained by the observation that proteasome inhibition prevents ubiquitin recycling, leading to depletion of free ubiquitin within 30 min to 3 hr (Melikova et al., 2006). Under these conditions, substrates that are transiently ubiquitinated and deubiquitinated will diminish in their ubiquitination in the face of limiting ubiquitin, whereas polyubiquitinated substrates that would have been degraded by the proteasome will accumulate.

These observations suggest that in cultured cells, CD4 and CD4-M1, which interact similarly with the Vpu-ligase complex, are both ubiquitinated. However, the slightly weaker-interacting CD4-M1 contains fewer ubiquitins and is not degraded. This is presumably due to constant DUB activity that limits chain growth. Inhibiting DUB activity should therefore preferentially reduce the levels of CD4-M1, thereby reducing its discrimination from CD4. Indeed, treatment of cells with the broadly acting DUB inhibitor PR-619 (Tian et al., 2011) reduced Vpu-mediated discrimination of CD4 from CD4-M1 by selectively reducing the levels of the latter (Figure 7C). Similar effects were seen with the CD4-Vpu-M1 pair. Prolonged treatment, although toxic to cells, led to nearly complete elimination of discrimination in the CD4-Vpu system (data not shown). Thus, as seen in vitro, optimal discrimination in cultured cells relies on DUB activity.

Conclusions and Perspective

Our in vitro, cellular, and in silico analysis of Vpu-mediated CD4 degradation shed light on general mechanisms underlying client discrimination, an event of critical importance in membrane protein quality and quantity control. We propose that discrimination with respect to eventual degradation is imparted by a combination of four parameters. First is the encounter of a potential client with the ligase complex, a prerequisite for ubiquitination. Second is the length of time this interaction is maintained, which would directly influence the number of ubiquitins that are added in each encounter (i.e., processivity). Third is the accessibility and activity of counteracting DUBs, which influences the length and dwell time of a ubiquitin chain. And fourth is downstream commitment events that determine client fate such as polyubiquitin-dependent engagement of the p97 complex for extraction (Wolf and Stolz, 2012), incorporation into COPII vesicles for ER export (Gillon et al., 2012), or achievement a configuration that markedly reduces ligase interaction. Uncommitted clients would re-enter the above cycle, with any delay in re-engaging the ligase resulting in rapid, unopposed deubiquitination to the ground state.

In the absence of DUB activity, our modeling and reconstitution studies suggest that discrimination would rely completely on large differences in client-ligase interaction. This is likely to be the case for degradation of soluble proteins from the lumen of ER, where access to the cytosolically disposed ligase activity is controlled by various upstream factors such as chaperones, adaptors, and glycosylation enzymes (Vembar and Brodsky, 2008). Discrimination for lumenal clients is therefore enforced by a chaperone network to triage clients between repeated folding attempts and delivery to a ligase, similar to reactions occurring during quality control in the cytosol (Buchberger et al., 2010).

By contrast, ligase access in the plane of the membrane is qualitatively different because the client and ligase are constrained to only two dimensions within a continuous compartment, and the catalytic activity of the ligase is accessible to the client without any topological rearrangement. Ligase-client interactions for membrane proteins may therefore be rather promiscuous and frequent, albeit relatively brief in most instances. It is in this regimen that discrimination relies on DUB activity to convert modest differences in interaction into larger differences in polyubiquitin acquisition and degradation. Our modeling and purified DUB experiments suggest that DUB activity, rather than a particular DUB, is sufficient for effecting discrimination. Nevertheless, it is plausible that individual quality-control pathways utilize different DUBs that are recruited to the site of ubiquitination (Blount et al., 2012). Indeed, many DUBs have been observed to interact with ligases for reasons that are unclear (Sowa et al., 2009).

The role for DUBs proposed here in controlling the generation of a degradation signal is worth distinguishing from downstream roles for DUBs. Once a degradation signal is generated and recruits ubiquitin binding proteins (such as the p97 complex; Wolf and Stolz, 2012), the substrate is committed for degradation. From this point, DUBs are thought to be involved in removing ubiquitins for threading through p97 (Ernst et al., 2009) and the proteasome (Ventii and Wilkinson, 2008). Given these multiple sites of action, it is not surprising that modulating deubiquitination in slightly different ways can have different outcomes for substrate degradation. For example, expression of a heterologous DUB free in the cytosol versus tethered to p97 affects different types of clients at different stages of the degradation process (Ernst et al., 2011). Similarly, a single client can be both stimulated and inhibited in its degradation depending on which DUB is knocked down (Sowa et al., 2009). These observations underscore the advantage of biochemically resolving a complex multistep process into individual reactions that can be mechanistically analyzed.

The appreciation of a role for DUBs in discrimination helps to explain otherwise puzzling aspects of quality control. For example, Vpu interacts sufficiently well with CD4 and βTrCP to not only coimmunoprecipitate with them, but also to retain them in the ER (Magadán et al., 2010; Magadán and Bonifacino, 2012). Despite these robust interactions and the fact that ubiquitination in the CD4-Vpu-SCF$^{\beta TrCP}$ complex occurs in mere

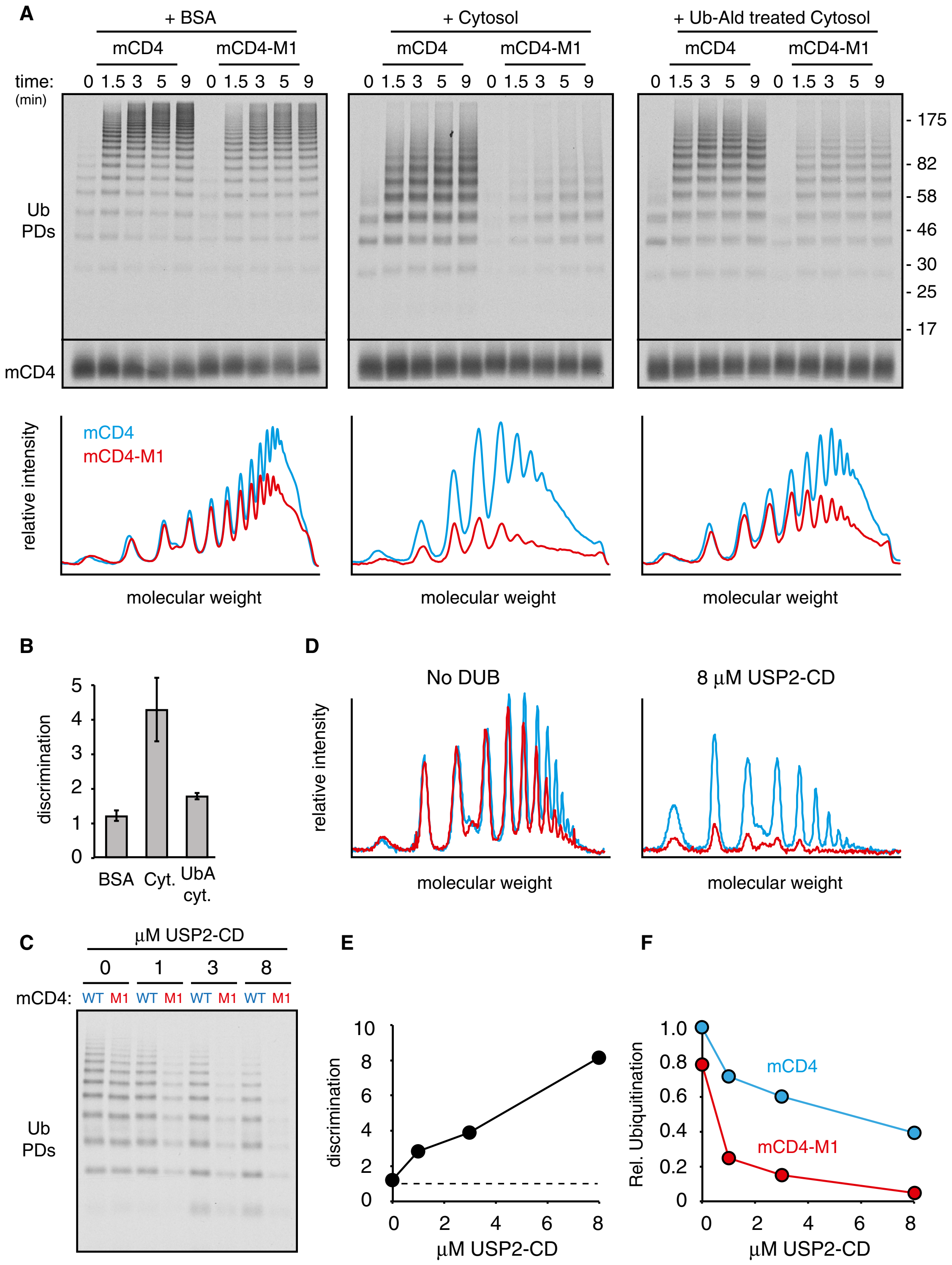

Figure 6. DUB Activity Contributes to Substrate Discrimination In Vitro

(A) The radiolabeled recombinant system was used to analyze mCD4 versus mCD4-M1 ubiquitination in the presence of BSA, HEK293 cell cytosol, or cytosol pretreated with ubiquitin-aldehyde. Note that ubiquitin-aldehyde only partially inhibits DUB activity (Figure S7B). The graph below the autoradiograph depicts the densitometry profiles of the 5 min samples.

(B) The ratio of polyubiquitination acquired on mCD4 divided by that acquired on mCD4-M1 in a parallel reaction is defined as discrimination. This value was determined for three experiments performed as in (A), quantified, and shown in the graph (mean ± SD).

(legend continued on next page)

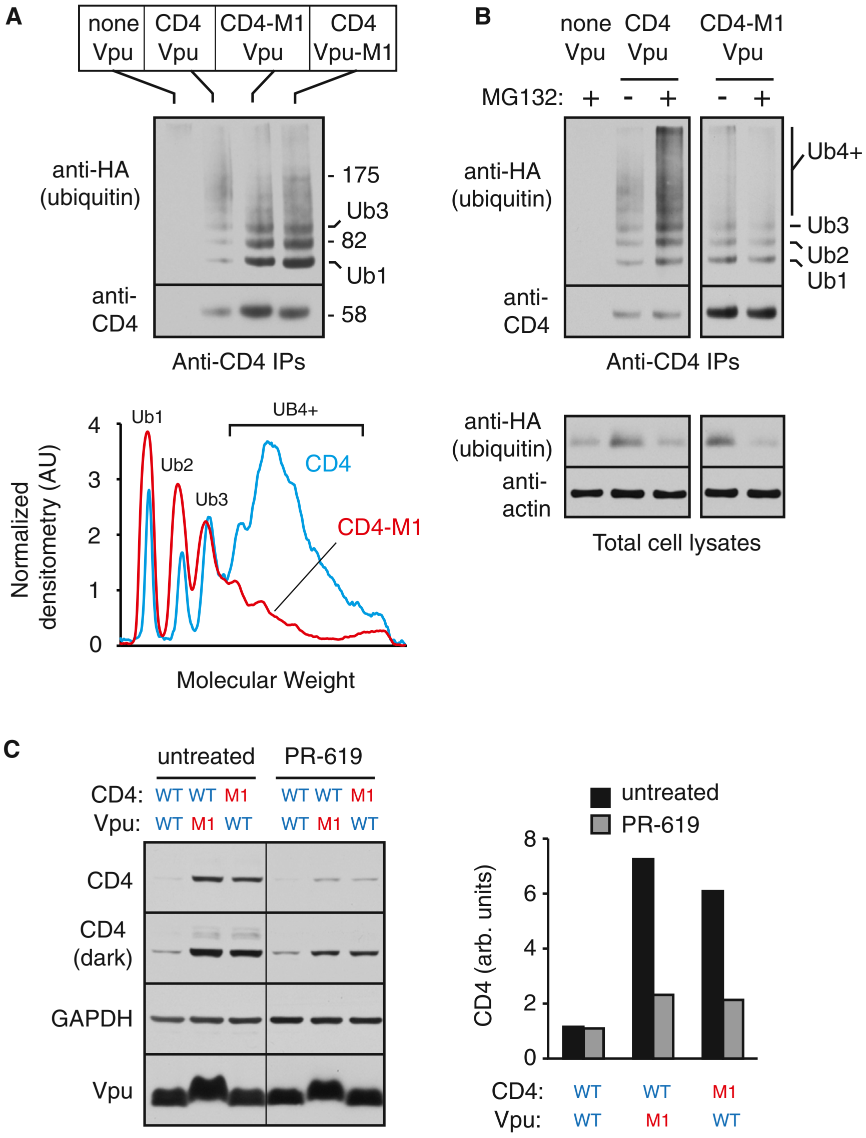

Figure 7. Evidence of a Role for DUBs in Substrate Discrimination in Cultured Cells

(A) Analysis of CD4 ubiquitination as in Figure 4D, but without proteasome inhibitor pretreatment. The positions of CD4 containing one and three ubiquitins are indicated. Below the blot is shown the ubiquitination profiles, normalized for the relative amount of total CD4, for the reactions containing CD4 versus CD4-M1.

(B) Analysis similar to (A) on cells pretreated without or with proteasome inhibitor (40 μM MG132 for 3 hr). The bottom panel shows immunoblots of total lysates, confirming that unconjugated ubiquitin levels are depleted several-fold after proteasome inhibition.

(C) Vpu-mediated degradation of CD4 in the absence and presence of the deubiquitinase inhibitor PR-619. Seven hours following transfection of the indicated plasmids, the cells were incubated for 13 hr without or with 25 μM PR-619 before harvesting and analysis by immunoblotting. Two exposures of the CD4 blot are shown. The results are quantified in the graph.

See also Tables S1 and S2.

seconds, CD4 degradation is remarkably slow ($t_{1/2}$ of 30 min or more). This can now be rationalized by the observation that very high DUB activity markedly reduces the proportion of substrates that acquire a polyubiquitin degradation signal with each ligase encounter, and the length of time that signal persists. This means that a large number of encounters are needed to get substantial degradation, explaining the relatively slow degradation of CD4 in cultured cells relative to its rapid polyubiquitination in vitro.

For substrates whose ligase interaction is marginally less stable, ubiquitins would be initially added at the same rate. However, the ligase would dissociate slightly earlier, allowing unopposed DUBs to rapidly deubiquitinate the substrate. The combination of less polyubiquitin production and shorter dwell time necessarily reduces opportunities for downstream steps in degradation, resulting instead in regeneration of the nonubiquitinated substrate. This provides a mechanism for achieving markedly different substrate half-lives for two clients with very similar ligase interactions. Although seemingly wasteful, the energy utilized for repeated de- and reubiquitination is employed to achieve substantial discrimination by essentially summing the outputs of numerous encounters.

This concept, a variation on kinetic proofreading, has been proposed to explain the order of degradation of key cell-cycle regulators (Rape et al., 2006). In that system, the APC ligase was shown to display differential processivity toward its clients due to differences in their interactions. The processivity differences were inversely related to their rate of degradation, providing an explanation for how proteins with modestly different

(C) Ubiquitination reactions of mCD4 versus mCD4-M1 in the radiolabeled recombinant system supplemented with the indicated concentrations of the recombinant USP2-CD deubiquitinase. Reaction time was 30 s, although similar results were seen at later time points as well.

(D) Densitometry analysis of the reactions from (C) without or with 8 μM USP2-CD.

(E) Discrimination plotted as a function of USP2-CD concentration derived from (C).

(F) Relative polyubiquitination efficiency of mCD4 and mCD4-M1 as a function of USP2-CD concentration. Values were normalized to the value observed for mCD4 in the absence of USP2-CD.

See also Figure S7 and Tables S1 and S2.

APC interaction are nevertheless discriminated effectively. Similar principles are used in signaling to convert modest differences in an input stimulus into large differences in output by exploiting multisite phosphorylation with competing dephosphorylation (Malleshaiah et al., 2010; Trunnell et al., 2011). By linking the output to a maximally phosphorylated state, competing dephosphorylation can effectively dampen output until a certain threshold input is reached.

Analogous principles utilizing multistep ubiquitination and competing deubiquitination may explain how two membrane proteins of very similar structure, such as Hmg2 in the presence of low versus high sterol, are decisively discriminated despite similar interactions with quality-control ligases. Reconstitution of such quality-control events in a tractable biochemical system represents an important future goal. We anticipate that many ligase interactions with membrane proteins will display modest differences between folded and misfolded versions. This is because ligases need to recognize a wide range of unrelated clients that are unlikely to share any uniform "degron," necessitating a rather broad sampling of potential clients using relatively weak interactions. Our findings suggest that achieving a wide dynamic range of degradation within a relatively narrow range of interactions involves exploiting the dynamic interplay between ubiquitination and deubiquitination, adding a previously unappreciated dimension to the mechanism of protein quality control.

EXPERIMENTAL PROCEDURES

Plasmids and Antibodies

Vpu- and CD4-derived constructs were modifications of published plasmids (Magadán et al., 2010) and described in Tables S1 and S2. The interacting coiled-coil segments used in Figure 3F have been described (Wang et al., 2011). Expression plasmids for $SCF^{\beta TrCP}$ and UbcH3 were from R. Deshaies and B. Schulman (Duda et al., 2008; Saha and Deshaies, 2008). Antibodies were from the following sources: anti-CD4 (Leica Microsystems), anti-Vpu (Magadán et al., 2010), anti-FLAG (Sigma-Aldrich), anti-Myc (Cell Signaling), anti-HA (Roche), anti-Actin (Abcam), anti-Cul1 (Invitrogen), anti-βTrCP (Invitrogen), anti-3F4 (Signet), anti-His6 (QIAGEN), anti-GAPDH (Sigma), and anti-CRT (Abcam).

Recombinant Proteins

Vpu and mCD4 were expressed in BL21 (DE3) pLysS cells and purified from inclusion bodies under denaturing conditions (with 6 M Urea) and the His tag removed by TEV protease. The Cul1/Rbx1 and βTrCP138/Skp1 subcomplexes were expressed, purified, and assembled as before (Li et al., 2005; Saha and Deshaies, 2008). Unmodified $SCF^{\beta TrCP}$ complex was used in Figures 3D, 3F, 4B, S4B, S4C, and S4E, whereas Nedd8-modified complex was used in all other experiments. UbcH3 was expressed and purified as before (Saha and Deshaies, 2008). Other proteins (Ube1, UbcH5c, various tagged ubiquitins, APPBP1/Uba3, UbcH12, Nedd8, and USP2 catalytic domain) were purchased from Boston Biochem.

Cell Culture Studies

HeLa, HEK293, and Flp-In 293 T-Rex cells (Invitrogen) were cultured in Dulbecco's modified Eagle's medium (DMEM) medium with 10% fetal bovine serum, 2 mM glutamine, and antibiotics. Transfections utilized Lipofectamine 2000 (Invitrogen). Pulse-chase analysis, ubiquitination analysis, IPs, SDS-PAGE, immunoblotting, and microsome isolation were as before (Furman et al., 2002; Magadán et al., 2010). Native IPs were performed on samples solubilized in lysis buffer containing 50 mM Tris-HCl (pH 7.5), 150 mM NaCl, 1% digitonin plus protease inhibitor cocktail (Roche).

In Vitro Translation and Reconstitution

Translation in rabbit reticulocyte lysate and protease protection assays has been described (Sharma et al., 2010; Fons et al., 2003). Reconstitutions were as before (Mariappan et al., 2011) and typically contained 5–1,000 ng recombinant Vpu, mCD4, 1% (w/v) DeoxyBigCHAP, and 200 μg unilamellar liposomes in a total volume of 100 μl. Detergent was removed with 40 mg Bio-beads and the proteoliposomes collected by centrifugation for use in ubiquitination assays. Lipid composition was typically 16:3.8:0.2 ratio of phosphatidyl-choline, phosphatidyl-ethanolamine, and rhodamine-PE. Ni-NTA lipid was included at 0.5 mol percent in Figure 3F.

In Vitro Ubiquitination

Ubiquitination reactions typically contained 2 mM ATP, 500 nM $SCF^{\beta TrCP}$ complex, and ubiquitin-charged E2s (5 μM UbcH3 and 0.2 μM UbcH5c) in the following buffer: 30 mM Tris-HCl (pH 7.5), 120 mM NaCl, 5 mM $MgCl_2$, and 2 mM DTT. The reaction was carried out at 25°C for various times. Samples were denatured in 2% SDS and boiled for 3 min prior to downstream analysis. His6-ubiquitin conjugates were purified using immobilized Co^{2+} resin.

Miscellaneous

SDS-PAGE used Tris-Tricine gels. Radioactive signals were detected on film for figures and phosphor screen for quantitation. Kinetic modeling employed KinTek explorer as described (Pierce et al., 2009) with details given in Figure S6C. For further details, please refer to Extended Experimental Procedures.

SUPPLEMENTAL INFORMATION

Supplemental Information includes Extended Experimental Procedures, seven figures, and two tables and can be found with this article online at http://dx.doi.org/10.1016/j.cell.2013.06.038.

ACKNOWLEDGMENTS

We thank R. Deshaies, B. Schulman, X. Yang, H. Ploegh, and V. Denic for reagents, M. Mariappan, J. Magadán, M.M. Babu, S. Shao, and M. Rodrigo-Brenni for discussions, and A. Sharma for technical support. This research was supported by the Intramural Research Program of the NIH, Intramural AIDS Targeted Antiviral Program of the NIH, and the Medical Research Council of the UK. Z.-R.Z. designed most of the experiments, performed all of the experiments, analyzed data, and helped draft the paper; R.S.H. conceived and guided the project, designed some of the experiments, analyzed data, and wrote the paper with input from Z.-R.Z.; J.S.B. provided advice, support, and comments on the paper.

Received: January 2, 2013
Revised: May 10, 2013
Accepted: June 20, 2013
Published: July 25, 2013

REFERENCES

Blount, J.R., Burr, A.A., Denuc, A., Marfany, G., and Todi, S.V. (2012). Ubiquitin-specific protease 25 functions in Endoplasmic Reticulum-associated degradation. PLoS One *7*, e36542.

Bour, S., Schubert, U., and Strebel, K. (1995). The human immunodeficiency virus type 1 Vpu protein specifically binds to the cytoplasmic domain of CD4: implications for the mechanism of degradation. J. Virol. *69*, 1510–1520.

Buchberger, A., Bukau, B., and Sommer, T. (2010). Protein quality control in the cytosol and the endoplasmic reticulum: brothers in arms. Mol. Cell *40*, 238–252.

Chen, M.Y., Maldarelli, F., Karczewski, M.K., Willey, R.L., and Strebel, K. (1993). Human immunodeficiency virus type 1 Vpu protein induces degradation of CD4 in vitro: the cytoplasmic domain of CD4 contributes to Vpu sensitivity. J. Virol. *67*, 3877–3884.

Chiti, F., and Dobson, C.M. (2006). Protein misfolding, functional amyloid, and human disease. Annu. Rev. Biochem. *75*, 333–366.

DeBose-Boyd, R.A. (2008). Feedback regulation of cholesterol synthesis: sterol-accelerated ubiquitination and degradation of HMG CoA reductase. Cell Res. *18*, 609–621.

Duda, D.M., Borg, L.A., Scott, D.C., Hunt, H.W., Hammel, M., and Schulman, B.A. (2008). Structural insights into NEDD8 activation of cullin-RING ligases: conformational control of conjugation. Cell *134*, 995–1006.

Ernst, R., Mueller, B., Ploegh, H.L., and Schlieker, C. (2009). The otubain YOD1 is a deubiquitinating enzyme that associates with p97 to facilitate protein dislocation from the ER. Mol. Cell *36*, 28–38.

Ernst, R., Claessen, J.H., Mueller, B., Sanyal, S., Spooner, E., van der Veen, A.G., Kirak, O., Schlieker, C.D., Weihofen, W.A., and Ploegh, H.L. (2011). Enzymatic blockade of the ubiquitin-proteasome pathway. PLoS Biol. *8*, e1000605.

Feldman, M., and van der Goot, F.G. (2009). Novel ubiquitin-dependent quality control in the endoplasmic reticulum. Trends Cell Biol. *19*, 357–363.

Feldman, R.M., Correll, C.C., Kaplan, K.B., and Deshaies, R.J. (1997). A complex of Cdc4p, Skp1p, and Cdc53p/cullin catalyzes ubiquitination of the phosphorylated CDK inhibitor Sic1p. Cell *91*, 221–230.

Fons, R.D., Bogert, B.A., and Hegde, R.S. (2003). Substrate-specific function of the translocon-associated protein complex during translocation across the ER membrane. J. Cell Biol. *160*, 529–539.

Furman, M.H., Ploegh, H.L., and Tortorella, D. (2002). Membrane-specific, host-derived factors are required for US2- and US11-mediated degradation of major histocompatibility complex class I molecules. J. Biol. Chem. *277*, 3258–3267.

Gardner, R.G., Shearer, A.G., and Hampton, R.Y. (2001). In vivo action of the HRD ubiquitin ligase complex: mechanisms of endoplasmic reticulum quality control and sterol regulation. Mol. Cell. Biol. *21*, 4276–4291.

Garza, R.M., Sato, B.K., and Hampton, R.Y. (2009). In vitro analysis of Hrd1p-mediated retrotranslocation of its multispanning membrane substrate 3-hydroxy-3-methylglutaryl (HMG)-CoA reductase. J. Biol. Chem. *284*, 14710–14722.

Gillon, A.D., Latham, C.F., and Miller, E.A. (2012). Vesicle-mediated ER export of proteins and lipids. Biochim. Biophys. Acta *1821*, 1040–1049.

Grove, D.E., Fan, C.Y., Ren, H.Y., and Cyr, D.M. (2011). The endoplasmic reticulum-associated Hsp40 DNAJB12 and Hsc70 cooperate to facilitate RMA1 E3-dependent degradation of nascent CFTRDeltaF508. Mol. Biol. Cell *22*, 301–314.

Hampton, R.Y. (2002). ER-associated degradation in protein quality control and cellular regulation. Curr. Opin. Cell Biol. *14*, 476–482.

Hassiepen, U., Eidhoff, U., Meder, G., Bulber, J.F., Hein, A., Bodendorf, U., Lorthiois, E., and Martoglio, B. (2007). A sensitive fluorescence intensity assay for deubiquitinating proteases using ubiquitin-rhodamine110-glycine as substrate. Anal. Biochem. *371*, 201–207.

Higy, M., Junne, T., and Spiess, M. (2004). Topogenesis of membrane proteins at the endoplasmic reticulum. Biochemistry *43*, 12716–12722.

Hirsch, C., Gauss, R., Horn, S.C., Neuber, O., and Sommer, T. (2009). The ubiquitylation machinery of the endoplasmic reticulum. Nature *458*, 453–460.

Isaacson, M.K., and Ploegh, H.L. (2009). Ubiquitination, ubiquitin-like modifiers, and deubiquitination in viral infection. Cell Host Microbe *5*, 559–570.

Ishikura, S., Weissman, A.M., and Bonifacino, J.S. (2010). Serine residues in the cytosolic tail of the T-cell antigen receptor alpha-chain mediate ubiquitination and endoplasmic reticulum-associated degradation of the unassembled protein. J. Biol. Chem. *285*, 23916–23924.

Lederkremer, G.Z., and Glickman, M.H. (2005). A window of opportunity: timing protein degradation by trimming of sugars and ubiquitins. Trends Biochem. Sci. *30*, 297–303.

Li, T., Pavletich, N.P., Schulman, B.A., and Zheng, N. (2005). High-level expression and purification of recombinant SCF ubiquitin ligases. Methods Enzymol. *398*, 125–142.

Malleshaiah, M.K., Shahrezaei, V., Swain, P.S., and Michnick, S.W. (2010). The scaffold protein Ste5 directly controls a switch-like mating decision in yeast. Nature *465*, 101–105.

Magadán, J.G., and Bonifacino, J.S. (2012). Transmembrane domain determinants of CD4 Downregulation by HIV-1 Vpu. J. Virol. *86*, 757–772.

Magadán, J.G., Pérez-Victoria, F.J., Sougrat, R., Ye, Y., Strebel, K., and Bonifacino, J.S. (2010). Multilayered mechanism of CD4 downregulation by HIV-1 Vpu involving distinct ER retention and ERAD targeting steps. PLoS Pathog. *6*, e1000869.

Margottin, F., Bour, S.P., Durand, H., Selig, L., Benichou, S., Richard, V., Thomas, D., Strebel, K., and Benarous, R. (1998). A novel human WD protein, h-beta TrCp, that interacts with HIV-1 Vpu connects CD4 to the ER degradation pathway through an F-box motif. Mol. Cell *1*, 565–574.

Mariappan, M., Mateja, A., Dobosz, M., Bove, E., Hegde, R.S., and Keenan, R.J. (2011). The mechanism of membrane-associated steps in tail-anchored protein insertion. Nature *477*, 61–66.

Meacham, G.C., Patterson, C., Zhang, W., Younger, J.M., and Cyr, D.M. (2001). The Hsc70 co-chaperone CHIP targets immature CFTR for proteasomal degradation. Nat. Cell Biol. *3*, 100–105.

Melikova, M.S., Kondratov, K.A., and Kornilova, E.S. (2006). Two different stages of epidermal growth factor (EGF) receptor endocytosis are sensitive to free ubiquitin depletion produced by proteasome inhibitor MG132. Cell Biol. Int. *30*, 31–43.

Meusser, B., and Sommer, T. (2004). Vpu-mediated degradation of CD4 reconstituted in yeast reveals mechanistic differences to cellular ER-associated protein degradation. Mol. Cell *14*, 247–258.

Nakatsukasa, K., Huyer, G., Michaelis, S., and Brodsky, J.L. (2008). Dissecting the ER-associated degradation of a misfolded polytopic membrane protein. Cell *132*, 101–112.

Nomaguchi, M., Fujita, M., and Adachi, A. (2008). Role of HIV-1 Vpu protein for virus spread and pathogenesis. Microbes Infect. *10*, 960–967.

Pierce, N.W., Kleiger, G., Shan, S.O., and Deshaies, R.J. (2009). Detection of sequential polyubiquitylation on a millisecond timescale. Nature *462*, 615–619.

Rape, M., Reddy, S.K., and Kirschner, M.W. (2006). The processivity of multiubiquitination by the APC determines the order of substrate degradation. Cell *124*, 89–103.

Rodrigo-Brenni, M.C., and Morgan, D.O. (2007). Sequential E2s drive polyubiquitin chain assembly on APC targets. Cell *130*, 127–139.

Saha, A., and Deshaies, R.J. (2008). Multimodal activation of the ubiquitin ligase SCF by Nedd8 conjugation. Mol. Cell *32*, 21–31.

Sato, B.K., Schulz, D., Do, P.H., and Hampton, R.Y. (2009). Misfolded membrane proteins are specifically recognized by the transmembrane domain of the Hrd1p ubiquitin ligase. Mol. Cell *34*, 212–222.

Schubert, U., and Strebel, K. (1994). Differential activities of the human immunodeficiency virus type 1-encoded Vpu protein are regulated by phosphorylation and occur in different cellular compartments. J. Virol. *68*, 2260–2271.

Schubert, U., Antón, L.C., Bacík, I., Cox, J.H., Bour, S., Bennink, J.R., Orlowski, M., Strebel, K., and Yewdell, J.W. (1998). CD4 glycoprotein degradation induced by human immunodeficiency virus type 1 Vpu protein requires the function of proteasomes and the ubiquitin-conjugating pathway. J. Virol. *72*, 2280–2288.

Shamu, C.E., Story, C.M., Rapoport, T.A., and Ploegh, H.L. (1999). The pathway of US11-dependent degradation of MHC class I heavy chains involves a ubiquitin-conjugated intermediate. J. Cell Biol. *147*, 45–58.

Sharma, A.S., Mariappan, M., Appathurai, S., and Hegde, R.S. (2010). In vitro dissection of protein translocation into the mammalian endoplasmic reticulum. Methods Mol. Biol. *619*, 339–363.

Singh, S.K., Möckel, L., Thiagarajan-Rosenkranz, P., Wittlich, M., Willbold, D., and Koenig, B.W. (2012). Mapping the interaction between the cytoplasmic domains of HIV-1 viral protein U and human CD4 with NMR spectroscopy. FEBS J. *279*, 3705–3714.

Skach, W.R. (2009). Cellular mechanisms of membrane protein folding. Nat. Struct. Mol. Biol. *16*, 606–612.

Sowa, M.E., Bennett, E.J., Gygi, S.P., and Harper, J.W. (2009). Defining the human deubiquitinating enzyme interaction landscape. Cell *138*, 389–403.

Tian, X., Isamiddinova, N.S., Peroutka, R.J., Goldenberg, S.J., Mattern, M.R., Nicholson, B., and Leach, C. (2011). Characterization of selective ubiquitin and ubiquitin-like protease inhibitors using a fluorescence-based multiplex assay format. Assay Drug Dev. Technol. *9*, 165–173.

Trunnell, N.B., Poon, A.C., Kim, S.Y., and Ferrell, J.E., Jr. (2011). Ultrasensitivity in the Regulation of Cdc25C by Cdk1. Mol. Cell *41*, 263–274.

Vembar, S.S., and Brodsky, J.L. (2008). One step at a time: endoplasmic reticulum-associated degradation. Nat. Rev. Mol. Cell Biol. *9*, 944–957.

Ventii, K.H., and Wilkinson, K.D. (2008). Protein partners of deubiquitinating enzymes. Biochem. J. *414*, 161–175.

Wang, F., Whynot, A., Tung, M., and Denic, V. (2011). The mechanism of tail-anchored protein insertion into the ER membrane. Mol. Cell *43*, 738–750.

Wolf, D.H., and Stolz, A. (2012). The Cdc48 machine in endoplasmic reticulum associated protein degradation. Biochim. Biophys. Acta *1823*, 117–124.

Wu, K., Kovacev, J., and Pan, Z.Q. (2010). Priming and extending: a UbcH5/Cdc34 E2 handoff mechanism for polyubiquitination on a SCF substrate. Mol. Cell *37*, 784–796.

EXTENDED EXPERIMENTAL PROCEDURES

Vpu-Mediated CD4 Degradation in Cultured Cells

HeLa or 293T cells were cotransfected with various combinations of CD4 and Vpu plasmids using Lipofectamine 2000. 18-24 hr after transfection, cells were washed with cold 1X PBS and then directly lysed in 2X protein sample buffer with boiling. The cell lysates were subject to immunoblotting as indicated in the Figure legends. For PR-619 treatment in Figure 7C, 25 μM PR-619 (obtained from LifeSensors) was added to the medium 7 hr after transfection and incubated for an additional 13 hr before harvesting the cells as above.

Ubiquitination Analysis in Cultured Cells

Cells were transfected with expression constructs for Vpu, CD4, and FLAG- or HA-tagged ubiquitin as indicated in the Figure legends and harvested 18-24 hr after transfection. Where indicated, the proteasome inhibitor MG132 was added to 40 μM for the specified period of time prior to harvesting. In Figure 4D, cells from individual wells of 6-well plates were lysed using 100 μl buffer containing 50 mM Tris-HCl, pH 7.4, 150 mM NaCl, 5 mM EDTA, 5 mM NEM, 10 mM iodoacetamide, 1% SDS, plus protease inhibitor cocktail and immediately heated to 95°C for 10 min. The resulting boiled lysates were centrifuged at maximum speed in a microcentrifuge to remove any aggregates and then diluted with 900 μl buffer containing 50 mM Tris-HCl, 150 mM NaCl, 5 mM NEM, 10 mM iodoacetamide, and 1% Triton X-100, followed by IP with 1.5 μl anti-CD4 monoclonal antibody (Leica Microsystems) for 2 hr or overnight. In Figures 7A and 7B, cells were directly lysed by resuspending in 500 μl RIPA buffer containing 50 mM Tris-HCl, pH 7.4, 150 mM NaCl, 5 mM EDTA, 5 mM NEM, 10 mM iodoacetamide, 0.2% sodium deoxycholate, 0.1% SDS, 1% Triton X-100, and protease inhibitor cocktail. Aggregates were removed by centrifugation at maximum speed in a microcentrifuge for 20 min. The supernatant was further centrifuged at 70,000 rpm (Beckman TLA100.3 rotor) for 30 min. The resulting supernatant was diluted with an equal volume of RIPA buffer without sodium deoxycholate and SDS and subjected to IP using 1.5 μl anti-CD4 antibody.

Native Immunoprecipitations

18-24 hr after transfection, cells were harvested and incubated with shaking for 20 min at 4°C in 500 μl lysis buffer containing 50 mM Tris-HCl, pH 7.5, 150 mM NaCl, 2% digitonin, and protease inhibitor cocktail,. The lysate was centrifuged at 30,000 rpm in a Beckman TLA100.3 rotor for 30 min. The resulting supernatant (500 μl) was diluted with 500 μl salt buffer containing 50 mM Tris-HCl, pH 7.5, 150 mM NaCl, and protease inhibitor cocktail. 1-3 μl antibody (or 10 μl antibody-conjugated agarose) was added and incubated for 2 hr or overnight, followed by incubation for 1 hr with Protein-A conjugated agarose to capture the antibodies. The beads were washed three times with 1 ml wash buffer (50 mM Tris-HCl, pH 7.5, 150 mM NaCl, 0.2% digitonin), and eluted with 50 μl 1.5x SDS-PAGE Sample Buffer. For IPs from proteoliposomes (Figure 3C), 40 μl liposome mixture was lysed in 1 ml buffer containing 50 mM Tris-HCl, 150 mM NaCl, and 1% Triton X-100. The resulting lysate was centrifuged at 30,000 rpm (Beckman TLA100.3 rotor) for 30 min and the supernatant was subjected to IP using anti-Vpu antibody (Magadán et al., 2010). In Figure 4B, 20 μl ubiquitination reaction was lysed with 500 μl buffer containing 50 mM Tris-HCl, 150 mM NaCl, 2% digitonin, followed by centrifugation at 60,000 rpm for 30 min. The sample was diluted with 500 μl salt buffer (50 mM Tris-HCl, 150 mM NaCl) and subjected to IP using anti-HA or IgG (control) antibody.

Stable-Cell Line Construction

Flp-In T-Rex-293 cells (Invitrogen) in 6-well plate was transfected using Lipofectamine with 300 ng pCDNA5/FRT/TO plasmids encoding HA tagged Vpu and 1700 ng pOG44 plasmid encoding Flp-recombinase. In parallel, a control group of cells were transfected with 300 ng pCDNA5/FRT/TO plasmid encoding Vpu and 1700 ng CFP plasmid. At 36-48 hr after transfection, cells were trypsinized and transferred into a 10-cm dish and cultured in DMEM medium plus 40 μg/ml hygromycin and 10 μg/ml blasticidin to select the positive cells. Medium and selection reagents were changed every 2 days. 10-15 days later, when the cells in control group were completely eliminated, the cells in the other flask were trypsinized, pooled, expanded, and used for experiments. In order to induce Vpu expression, 20-100 ng/ml doxycycline was added to medium for 20-48 hr.

Microsome Preparation

Canine pancreatic microsomes were prepared and used as described (Sharma et al., 2010). In order to isolate microsomes from cultured cells, Vpu inducible cells were cultured in 10-cm dishes in media without hygromycin for 3-5 generations. This was essential to completely eliminate any hygromycin contamination that otherwise inhibits translation. When the cells were 90% confluent, they were transferred to a 15-cm dish in the media without hygromycin. Doxycycline (20-100 ng/ml) was added to the media and Vpu expression was induced for 48 hr, with a media change after 24 hr. With the cells at ~95% confluency, they were washed while on the dish with cold PBS and the cells from each dish collected by pipetting in 5 ml PBS. The pooled cells from 8 dishes were harvested by centrifugation at 1500 x g for 10 min, and the supernatant was removed thoroughly to leave a cell pellet of ~1 ml. The cells were either used directly or flash frozen in liquid nitrogen and stored at −80°C for later use. The 1 ml cell pellet was resuspended in 2 ml hypotonic buffer (Buffer A, 10 mM HEPES, pH 7.4, 250 mM Sucrose, 2 mM $MgCl_2$, 1 x protease inhibitor cocktail) and incubated for several minutes on ice before homogenization by repeated passage through 19-, 23- and 25- gauge needles using a 3-ml syringe.

Cells were lysed with ~95% efficiency as monitored by microscopy. The cell lysate was diluted with 3 ml buffer A and unbroken cells and nuclei were pelleted by centrifugation at 3,716 x g for 30 min. The supernatant was collected and recentrifuged to remove nuclei thoroughly. The resulting supernatant was centrifuged at 75,000 x g (33,000 rpm, Beckman MLA-80 rotor) for 60 min at 4°C. The resulting pellet was resuspended and gently homogenized in 10 mM HEPES, pH 7.4, 0.25 M sucrose, 1 mM $MgCl_2$ and 0.5 mM DTT. Microsomes were frozen in liquid nitrogen and stored at −80°C. Prior to use in translation reactions, microsomes from HEK293 cells were treated with nuclease to remove endogenous mRNA. Briefly, $CaCl_2$ was added to 0.33 mM, micrococcal nuclease was added to 50 units/ml, and incubated at 25°C for 5 min. The reaction was terminated with 0.66 mM EGTA (final concentration) and transferred to ice.

Cytosol Preparation

HEK293T cells or HeLa cells were harvested by scraping in the presence of ice cold PBS. The cell pellet from eight 15-cm dishes (~1 ml packed volume) was resuspended with an equal volume of hypotonic buffer containing 10 mM HEPES, pH 7.4, 0.25 M sucrose, 1 mM $MgCl_2$ and 1 mM DTT plus protease inhibitor cocktail. After incubation for several minutes on ice, the mixture was homogenized by repeated passage through 23 and 25 gauge needles. NaCl was then added to the lysate to a final concentration 110 mM. The cytosol fraction was attained by differential centrifugation: 3,000 x g for 15 min, 16,100 x g for 12 min, and 70,000 rpm (Beckman TLA100.3) for 30 min. The concentration of cytosol was determined by Bradford method and was typically 15-20 mg/ml.

Recombinant Protein Production in *E. coli*

Expression of Vpu, mCD4, VpuCyto, mCD4Cyto, or coiled/coil-Vpu/CD4-Cyto in *E. coli* BL21(DE3)pLysS cells from the pRSETA or pET-Duet vector was induced with 1 mM IPTG at 37°C for 4 hr, or 0.4 mM IPTG at 18°C for 16 hr. The His-tagged membrane proteins Vpu and mCD4 were solubilized and purified under denaturing conditions (with 50 mM Tris-HCl, 0.3 M NaCl, 1% CHAPS, 6 M urea) using 1 ml Hi-Trap chelating HP affinity column from GE Healthcare, with immobilized Co^{2+} and Ni^{2+}, respectively. The protein was refolded on the column by washing extensively with renaturing buffer without urea (50 mM Tris-HCl, 0.3 M NaCl, 1% CHAPS) and eluted with 0.3 M imidazole in renaturing buffer before dialysis against renaturing buffer to remove imidazole. The His tag was cleaved by TEV protease and the TEV was removed by passing through a column of immobilized Ni^{2+}. His-tagged VpuCyto, CD4Cyto, and the Vpu-CD4 coiled coil complex were purified from the 40,000 x g supernatant of *E. coli* lysate prepared in a buffer containing 50 mM Tris-HCl, and 0.3 M NaCl using 1 ml Hi-Trap chelating Sepharose HP affinity columns. VpuCyto utilized immobilized Co^{2+}, while the other two utilized immobilized Ni^{2+}. The concentrations of these proteins were determined by measuring absorbance at 280 nm and Bradford method.

Phosphorylation of Vpu, VpuCyto, and Coiled/Coil-Vpu/CD4-Cyto

Purified Vpu, VpuCyto or coiled/coil-Vpu/CD4-Cyto was phosphorylated by incubation with CK2 (New England Biolabs). A typical 5 μl reaction contained 100 units of CK2 and 1 μg recombinant protein in 50 mM HEPES, pH 7.5, 0.3 M NaCl, 10 mM $MgCl_2$, 50 mM KCl, and 2 mM ATP (plus 0.5% DBC or CHAPS for purified Vpu with transmembrane domain). The reaction was performed at 25°C for 10 min. This resulted in fully phosphorylated Vpu, VpuCyto and coiled/coil-Vpu/CD4-Cyto. The mixture was used directly in reconstitutions without further purification since the soluble CK2 is effectively removed when proteoliposomes are recovered by centrifugation (see below).

Experiments in Crude Microsomes

The experiments in Figures 2D, 4E, S2B, S2C, and S5D were performed on mCD4 substrates produced by in vitro translation and inserted into crude ER microsomes as follows. In vitro translations were performed using rabbit reticulocyte lysate (RRL) as described previously (Sharma et al., 2010) but contained microsomes isolated from different Vpu-expressing or control HEK293 cells. Following translation, the microsomes were isolated by one of three ways. In Figure 4E and S5D, the translation reactions were diluted 4-fold in salt buffer (50 mM HEPES, pH 7.4, 100 mM KAc, 1 mM $MgCl_2$) and sedimented using a Beckman TLA100.3 rotor at 70,000 rpm for 10 min. For Figures S2B and S2C, the translation reactions were layered on a sucrose cushion (containing 0.5 M sucrose, 100 mM KAc, 2 mM $MgCl_2$) and sedimented using a Beckman TLA100.3 rotor at 70,000 rpm for 10 min. For Figure 2D, samples were chilled on ice and adjusted to high salt conditions (50 mM HEPES, 0.8 M KAc, 1 mM $MgCl_2$) in a final volume of 100-200 μl and incubated on ice for 10 min. The samples were layered onto a 500 μl sucrose cushion containing 50 mM HEPES, pH 7.4, 0.8 M KAc, 250 mM sucrose, 1 mM $MgCl_2$ and centrifuged using a Beckman TLA100.3 rotor at 70,000 rpm for 10 min. The resulting pellet was resuspended with high salt buffer and centrifuged again. The final microsome pellets from each of the above isolation procedures were then resuspended in 50 mM HEPES, pH 7.4, 100 mM KAc, 250 mM sucrose, 1 mM $MgCl_2$, and used in ubiquitination reactions as described below. Note that the three methods give very similar results. Nevertheless, salt washing does reduce background ubiquitination by effectively removing any noninserted mCD4 substrate bound to the surface of the microsomes. Noninserted mCD4 seems to contribute background ubiquitination via endogenous ligases that recognize it as a misfolded protein. This is why the ubiquitination reactions in Figure 2D shows no background in the absence of phospho-Vpu, while the experiments in Figure S2 show some background.

Liposome Preparation

Liposomes were prepared as described before (Mariappan et al., 2011). In brief, lipids in organic solvent were mixed in glass vials at the desired ratio [typically 16:3.8:0.2 of phosphatidyl-choline (PC; Avanti Cat no. 840051C), phosphatidyl-ethanolamine (PE; Avanti Cat no. 810332C), and rhodamine-PE (Avanti Cat no. 81058P)], adjusted to 10 mM DTT, dried in a speedvac, and resuspended in 10 mM HEPES, pH 7.4, 15% glycerol. The suspension was adjusted to 20 mg/ml lipids and extruded (using an extruder from Avanti) through polycarbonate membranes with pore size 1 μm, then 0.2 μm, at 60°C, until the mixture turned from cloudy to transparent.

Preparation of Proteoliposomes Containing Recombinant Proteins

Purified mCD4 and/or Vpu were adjusted to 100 ul in reconstitution buffer (50 mM HEPES, pH 7.4, 500 mM KAc, 2 mM $MgCl_2$, 2 mM DTT, 250 mM sucrose, 1% DBC), incubated for 10 min on ice, and centrifuged at 72,000 rpm (Beckman TLA100.3 rotor) for 30 min to remove any insoluble material. The resulting supernatant was supplemented with 10 μl preformed liposomes (see above) and incubated for 30-60 min on ice. The 110 μl mixture was added to 40-45 mg Biobeads SM2 (BioRad) and incubated overnight to remove detergent. The sample was separated from the Biobeads and diluted with 500 μl cold water plus 2 mM DTT. The proteoliposomes were pelleted by centrifugation at 72,000 rpm for 45 min using Beckman rotor TLA100.3. The supernatant was removed carefully and discarded. The proteoliposome pellets were used in ubiquitination assays as described below.

Preparation of Proteoliposomes Containing Radiolabeled Substrate

In vitro translation of mCD4 or mCD4-M1 was performed in RRL containing ^{35}S-Methionine and canine pancreatic ER microsomes. Note that the canine microsomes do not contain Vpu, and therefore do not contain tightly bound $SCF^{\beta TrCP}$. Following translation, the microsomes were isolated by sedimentation through a sucrose cushion and resuspended in the original translation volume of reconstitution buffer (50 mM HEPES, pH 7.4, 500 mM KAc, 2 mM $MgCl_2$, 2 mM DTT, 250 mM sucrose, 1% DBC). To the solubilized sample was added 5-1000 ng purified recombinant Vpu (or mutants) prephosphorylated with CK2 as described above. After incubation for 5-10 min on ice, the sample was centrifuged at 72,000 rpm (Beckman TLA100.3 rotor) for 30 min to remove any insoluble material. The resulting supernatant was supplemented with 10 μl pre-formed liposomes (see above) and incubated for 30-60 min on ice. The 110 μl mixture was added to 40-45 mg Biobeads SM2 (BioRad) and incubated overnight to remove detergent. The sample was separated from the Biobeads and diluted with 500 μl cold water plus 2 mM DTT. The proteoliposomes were pelleted by centrifugation at 72,000 rpm for 45 min using Beckman rotor TLA100.3. The supernatant was removed carefully and discarded. The proteoliposome pellets were used in ubiquitination assays as described below. Analysis of the proteoliposomes showed that cytosolic proteins from the original in vitro translation extract were undetectable, and that microsomal proteins were removed by at least 95%. Any remaining contamination was inert with respect to ubiquitination or deubiquitination of mCD4 as determined in independent assays. In particular, no ubiquitination was observed in the absence of the Vpu-SCF complex. Furthermore, after ubiquitination by the SCF, the ubiquitinated products were stable and no deubiquitination was observed. Finally, we found that adding an affinity step to the above procedure to purify the mCD4 via its His6 tag gave identical results as without this extra step.

In Vitro Ubiquitination Reactions

The pelleted microsomes or proteoliposomes prepared as described above were resuspended in 10 μl 2X ubiquitination buffer (60 mM Tris-HCl, pH 7.5, 200 mM NaCl, 10 mM $MgCl_2$, 4 mM DTT), plus an additional 2 μl H_2O. The mixture was homogenized carefully by pipetting, resulting in ~14 μl liposome suspension, which was then adjusted with 2 μl ATP (20 mM), 1 μl βTrCP/Skp1 (20 μM), 1 μl Cul1/Rbx1 (20 μM), and either 2 μl H_2O or Neddylation reagents [1 μl APPBP1/Uba3 (5 μM), 0.5 μl UbcH12 (115 μM), 0.5 μl Nedd8 (225 μM)]. The 20 μl mixture was then incubated at 25°C for 5 min to allow assembly of the $SCF^{\beta TrCP}$ on phospho-Vpu and Neddylation of Cul1 (validated by the shift of the Cul1C band in Coomassie stained gel). For reactions in Figure 6C, USP2 catalytic domain (USP2CD) was added at this point. This is termed "mixture A."

In parallel, 20 μl mixture B was made by mixing 9.64 μl H_2O, 2 μl 10X Ubiquitination buffer (300 mM Tris-HCl, pH 7.5, 1 M NaCl, 20 mM DTT, 50 mM $MgCl_2$), 2 μl ATP (20 mM), 1.6 μl Ube1 (5 μM), 4 μl UbcH3 (52 μM), 0.16 μl UbcH5c (50 μM), and 0.6 μl tagged ubiquitin (1 mM). Mixture B was incubated at 25°C for 3 min to achieve fully ubiquitin-charged UbcH3 and UbcH5c (Pierce et al., 2009). The mixtures A and B were combined to initiate the ubiquitin conjugation reaction.

For in vitro ubiquitination in the presence of BSA, cytosol, or ubiquitin-aldehyde treated cytosol (Figure 6A and S7A), the recipe for ubiquitination reactions was slightly modified to make space for cytosol in the reaction. The proteoliposome pellet was resuspended directly in 20 μl cytosol or BSA (18 mg/ml typically), plus 0.2 μl okadaic acid (119 μM, Sigma) to inhibit protein phosphatase activity in the cytosol. The following reagents were added to the suspension: 1.5 μl 20X energy regenerating system (20X ERS: 20 mM ATP, 20 mM GTP, 0.8 mg/ml creatine kinase, and 200 mM creatine phosphate), 1 μl βTrCP/Skp1 (20 μM), and 1 μl Cul1/Rbx1 (20 μM) plus 2 μl Neddylation system (1 μl APPBP1/Uba3 (5 μM), 0.5 μl UbcH12 (115 μM) and 0.5 μl Nedd8 (225 μM)). The resulting 25 μl mixture was incubated at 25°C for 5 min to achieve complete Neddylation of Cul1C. To make fully charged E2s, the recipe for 15 μl mixture B is: 5.1 μl H_2O, 2 μl 10X Ubiquitination buffer (300 mM Tris-HCl, pH 7.5, 1 M NaCl, 20 mM DTT, 50 mM $MgCl_2$), 1.5 μl 20X ERS, 1.6 μl Ube1 (5 μM), 4 μl UbcH3 (52 μM), 0.16 μl UbcH5c (50 μM), and 0.6 μl tagged ubiquitin (1 mM). As above, mixtures A and B were combined to initiate the ubiquitin conjugation reaction.

For the experiments in Figures 2D, S2B, and S2C, the E2 enzymes were not "precharged" but simply included in the reaction. The ubiquitination reactions contained 40 mM Tris-HCl, pH 7.5, 100 mM NaCl, 5 mM $MgCl_2$, 1X ERS, 1 mM DTT, 10 eq RM. 0.1 μM Ube1,

0.2 μM UbcH3, 0.1 μM UbcH5c and 10 μM His-tagged ubiquitin. The samples were incubated at 25°C for 1 hr. All ubiquitination reactions were stopped by denaturation with 1% SDS, diluted with Triton X-100 and subjected to pull down using immobilized Co^{2+}.

In Vitro Crosslinking

Proteoliposomes containing recombinant phospho-Vpu and radiolabeled mCD4 or mCD4-M1 from a typical 110 μl reconstitution (see above) were isolated by centrifugation and resuspended thoroughly in 10 μl crosslinking buffer (20 mM HEPES, pH 7.4, 100 mM NaCl, 1 mM EDTA, 250 mM Sucrose). 1 μl recombinant βTrCP/Skp1 protein complex (20 μM stock) was added and incubated with proteoliposomes for 10 min on ice to facilitate binding. Samples were then diluted by adding 68.2 μl crosslinking buffer to achieve 79.2 μl volume. 0.8 μl BMH (Pierce, 30 mM in DMSO) was added to a final concentration of 300 μM. Incubation was performed at room temperature for 30 min. The reaction was quenched with 10 mM DTT, and the samples were fully denatured by adjusting to 100 mM Tris, 1% SDS and boiling briefly.

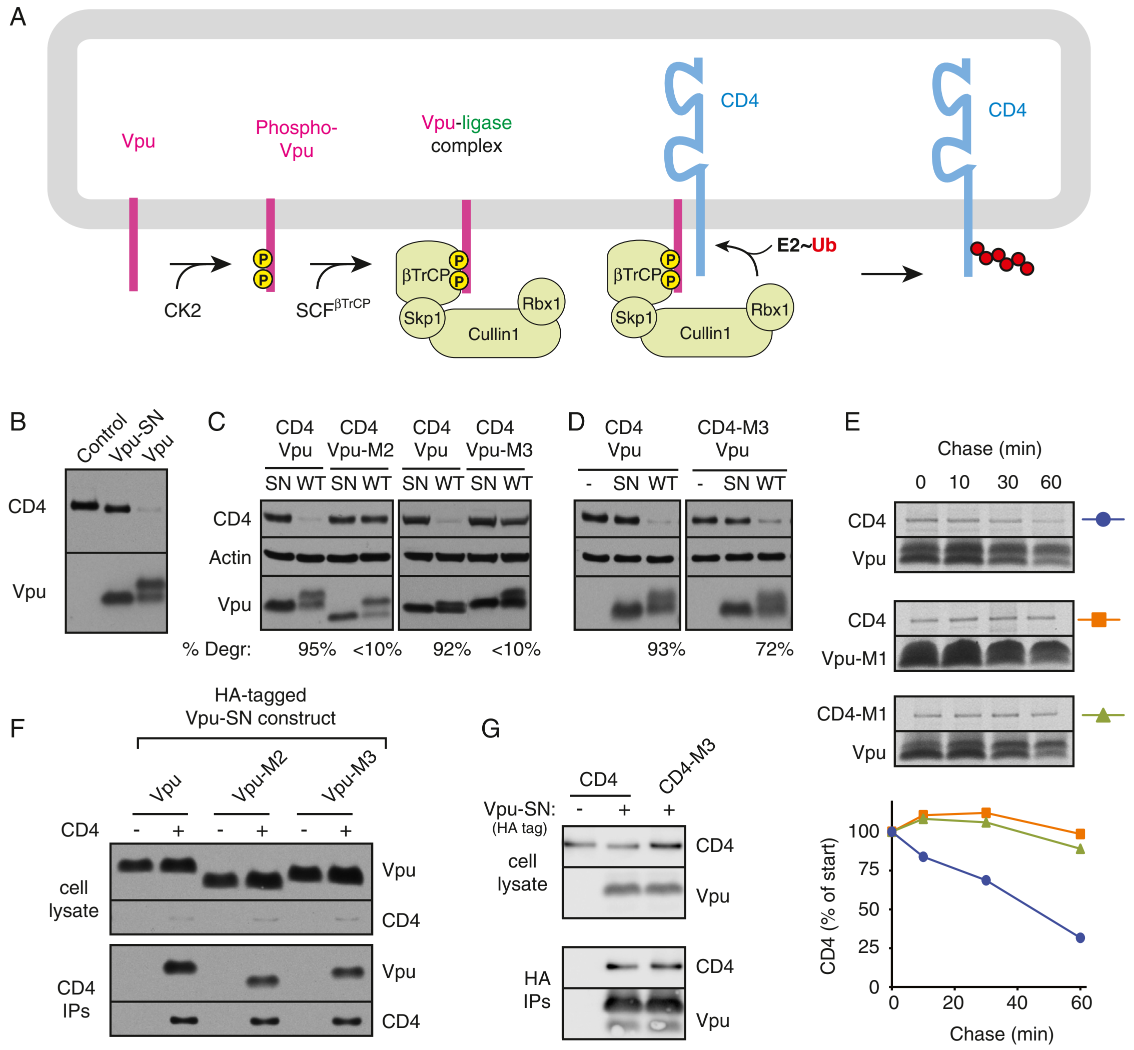

Figure S1. Characterization of Vpu-Mediated CD4 Degradation, Related to Figure 1

(A) Schematic diagram of steps and components involved in Vpu-mediated CD4 ubiquitination.

(B) CD4 was cotransfected with Vpu, Vpu-SN, or empty vector (control) and analyzed for Vpu and CD4 levels. Note that Vpu is phosphorylated (evident by its slower migration on the gel), whereas the SN mutant is not.

(C and D) Analysis of various Vpu-CD4 pairs for degradation of CD4. After cotransfection, the cell lysates were analyzed for CD4, Vpu, and Actin (a loading control) by immunoblotting. In each pair, the SN mutant serves as a negative control since this completely precludes ligase recruitment and CD4 degradation (e.g., B). The percent degraded (normalized to the respective SN mutant control) is indicated below the blots.

(E) Pulse-chase analysis of CD4 or CD4-M1 degradation in the presence of Vpu or Vpu-M1. Pulse labeling with ^{35}S-Methionine was for 5 min, and chased with unlabeled Methionine for up to 60 min. CD4 and Vpu were recovered by IP and analyzed by SDS-PAGE and autoradiography. The results were also quantified by phosphorimaging and displayed in the graph below.

(F) Analysis of CD4-Vpu interactions by co-IP. The indicted HA-tagged Vpu constructs (each containing the SN mutation to prevent CD4 degradation) were co-expressed with CD4 (or a control plasmid) and analyzed by co-IP using anti-CD4. The total lysates (top) and CD4 IPs were blotted for Vpu and CD4.

(G) HA-tagged Vpu-SN (or a control plasmid) was cotransfected with either CD4 or CD4-M3 and analyzed for interaction by anti-HA IPs. Total lysates and HA IPs were analyzed by blotting for CD4 and Vpu. Note that M1, M2, and M3 refer to versions of CD4 or Vpu containing heterologous or mutant TMDs in place of their respective native TMDs (see Tables S1 and S2). Vpu-M1 contains the mutations I17F/V21F/V25L, Vpu-M2 contains the glycophorin A TMD, and Vpu-M3 contains the VSVG TMD. CD4-M1 contains the glycophorin A TMD, CD4-M2 contains the VSVG TMD, and CD4-M3 contains the Vpu TMD (to allow a homotypic interaction with Vpu).

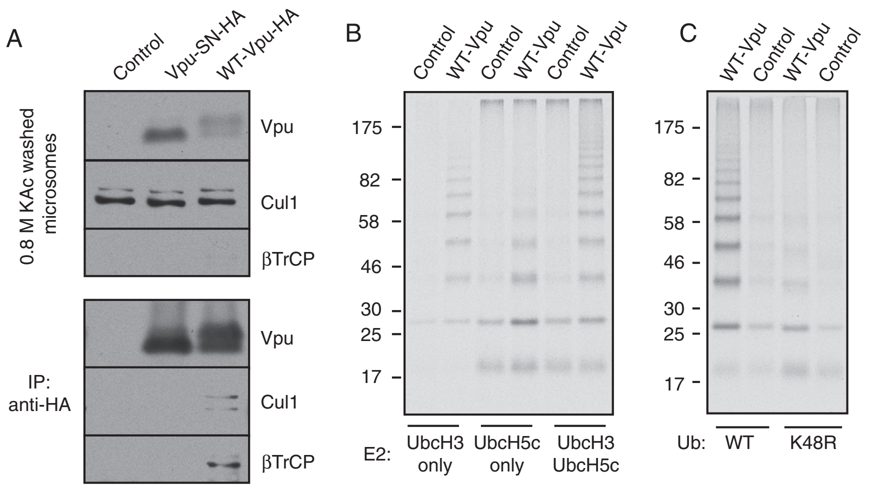

Figure S2. Features of mCD4 Ubiquitination on Microsomes by Vpu, Related to Figure 2

(A) Microsomes were isolated from HEK293 cells ('control'), HEK293 cells expressing HA-tagged wild-type Vpu ('WT-Vpu-HA'), or HEK293 cells expressing Vpu-SN ('Vpu-SN-HA') and subjected to high salt wash with 0.8 M KAc. The salt-washed microsomes were analyzed directly (top) or solubilized and subjected to anti-HA IPs (to recover the HA-tagged Vpu) prior to immunoblotting. Note that βTrCP was undetectable on total microsomes, but detected upon enrichment by co-IP with Vpu.

(B) mCD4 was in vitro translated in the presence of either control or Vpu-expressing microsomes from HEK293 cells. The microsomes were isolated and subjected to ubiquitination assays containing His6-ubiquitin, E1, ATP, and the indicated E2 enzymes. The ubiquitinated products were isolated using immobilized Co^{2+} and analyzed by SDS-PAGE and autoradiography.

(C) Ubiquitination assay as in B, but containing either wild-type His6-ubiquitin, or His6-ubiquitin containing a K48R mutation. Note that polyubiquitination is sharply reduced by the K48R mutant ubiquitin.

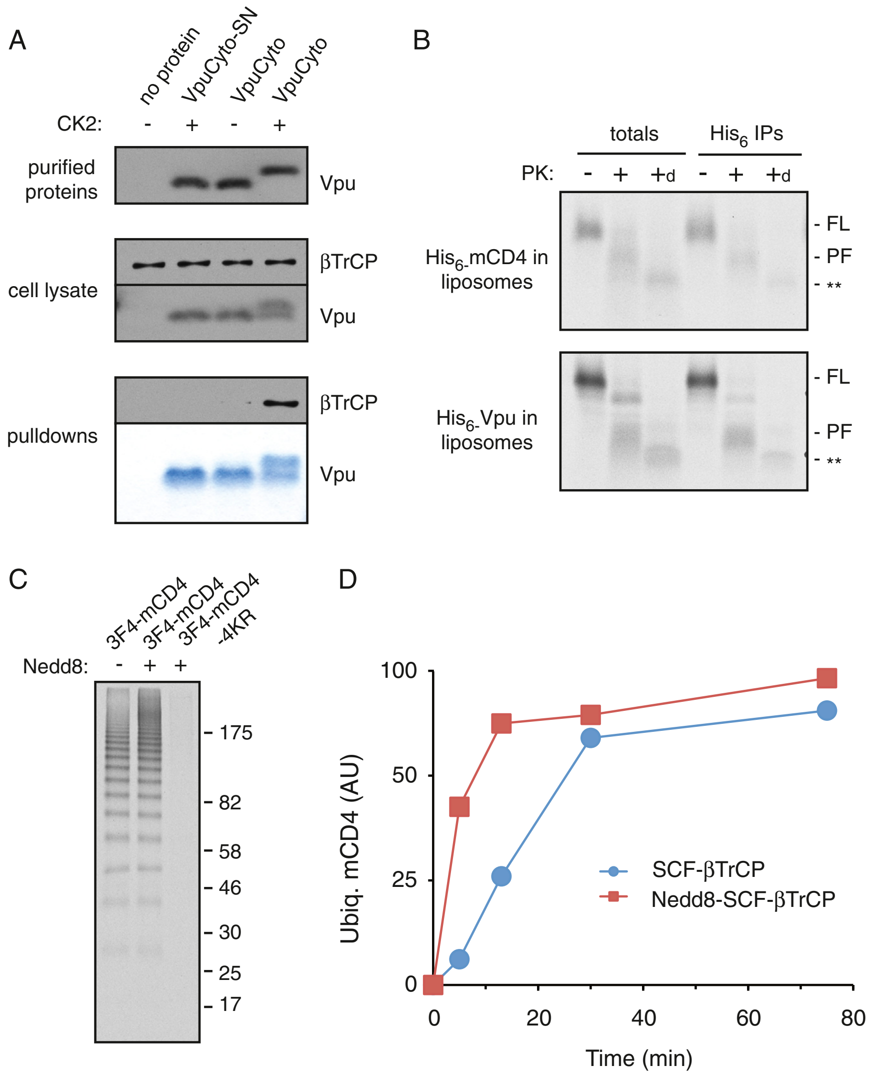

Figure S3. Vpu Interactions, Topology, and Activity in Proteoliposomes, Related to Figure 3

(A) His6-tagged cytosolic domains of Vpu or Vpu-SN were expressed and purified from *E. coli* and incubated with CK2 as indicated. An aliquot was analyzed by immunoblotting (top) to verify efficient phosphorylation (as evidenced by a shift on the gel). The purified proteins were incubated with HeLa cytosol containing overexpressed βTrCP1 and subjected to pulldown using immobilized Co^{2+}. The cell lysate (containing the added purified proteins) and pulldowns were analyzed by SDS-PAGE and immunoblotting or Coomassie staining. βTrCP is selectively pulled down in a phosphor-Vpu-dependent manner.

(B) Liposomes were reconstituted with radiolabeled His6-tagged mCD4 or Vpu. They were either left untreated, digested with proteinase K (PK), or PK in the presence of 0.5% Triton X-100 (indicated by subscript ‘d’). The samples were either analyzed directly, or immunoprecipitated using an antibody against the His6 tag. The positions of full-length (FL) and primary protease-protected N-terminal fragments (PF) are indicated. ‘**’ indicates a protease-resistant fragment. Note that the heterogeneous bands seen with PK digestion probably arise from variations in precisely where within the cytosolic tails PK cuts.

(C) Radiolabeled mCD4 or mCD4-4KR (in which the four lysines in the cytosolic tail of CD4 are mutated to arginines) isolated from in vitro translation reactions was coreconstituted with recombinant Vpu into liposomes. The resuspended liposome sample was subjected to ubiquitination reactions using purified $SCF^{\beta TrCP}$ that had or had not been modified with Nedd8 as indicated. The ubiquitinated products (isolated by pulldown via the tagged ubiquitin) of the 30 min reaction are shown.

(D) Time course of ubiquitination reactions as in C quantified by phosphorimaging shows that Neddylation of SCF improves its reaction speed.

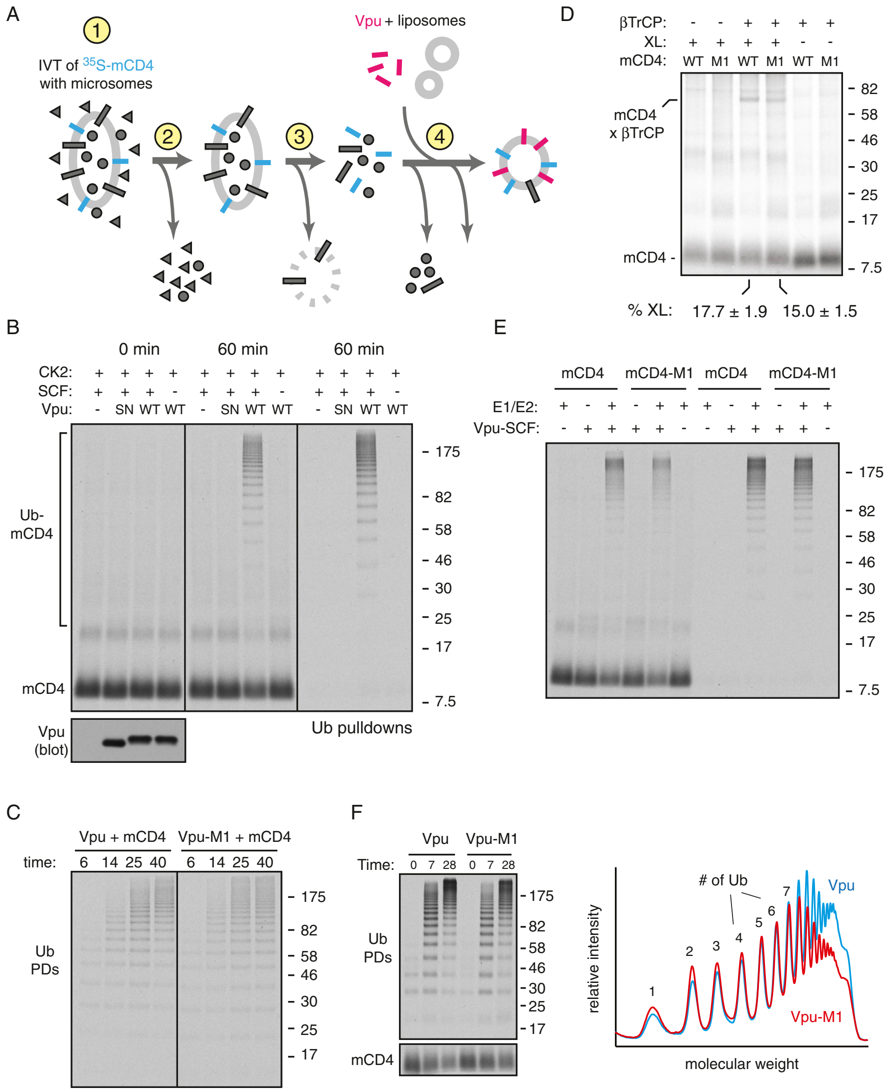

Figure S4. Minimal Substrate Discrimination Is Observed in the Reconstituted System, Related to Figure 4

(A) Schematic depicting the procedure for preparing proteoliposomes containing radiolabeled mCD4 and recombinant Vpu. The starting in vitro translation reaction used to produce ^{35}S-methionine-labeled mCD4 contains cytosolic proteins and microsomal proteins. These are progressively removed during the reconstitution procedure such that cytosolic proteins are not detectable in the final proteoliposomes and microsomal proteins are removed to more than 95%.

(B) Radiolabeled in vitro produced mCD4 was coreconstituted into liposomes using the procedure in A with either nothing else, recombinant Vpu, or Vpu-SN. The proteoliposomes were then treated with CK2 to phosphorylate Vpu, and subjected to ubiquitination reactions containing or lacking SCF$^{\beta TrCP}$ as indicated. The samples were analyzed by autoradiography to detect mCD4 or immunoblotting to detect Vpu. An aliquot of the samples were also subjected to pulldowns via the His6-tag on ubiquitin to visualize the ubiquitinated products. Note that ubiquitinated products (representing approximately 60% of mCD4) were only seen when the reaction contained both phospho-Vpu and SCF$^{\beta TrCP}$. This indicates that no other ligase activity capable of ubiquitinating mCD4 is present in this reconstituted system.

(C) Radiolabeled mCD4 was produced as in A, except an additional Co^{+2} affinity step was included to further purify the substrate prior to reconstitution into proteoliposomes with Vpu or Vpu-M1. Shown are the ubiquitinated products at different times from a reaction similar to that in B.

(D) Proteoliposomes containing recombinant phosphorylated Vpu and the radiolabeled substrates mCD4 (or mCD4-M1) were produced as in B and incubated with recombinant βTrCP/Skp1 complex where indicated. The samples were treated with the cysteine-reactive crosslinker BMH as indicated, quenched with DTT, and analyzed by SDS-PAGE and autoradiography to detect mCD4 and mCD4-M1. Note that recombinant Vpu does not have cysteine residues and therefore does not participate in crosslinking. The position of the substrate crosslink to βTrCP is indicated. Crosslinking efficiencies for mCD4 and mCD4-M1 are nearly the same (mean ± SD; n = 6).

(E) An experiment similar to B was performed with mCD4 versus mCD4-M1 as the substrate. Note that ubiquitination is more than 50% efficient, completely dependent on the Vpu-SCF complex, and very similar between mCD4 and mCD4-M1.

(F) Radiolabeled mCD4 coreconstituted with either Vpu or Vpu-M1 in liposomes was subjected to ubiquitination. Aliquots at different time points were analyzed by ubiquitin pulldowns (top) or directly for mCD4 levels (bottom). The traces at the right are densitometry profiles of the two reactions at 7 min, illustrating a selective reduction of highly poly-ubiquitinated products selective with Vpu-M1.

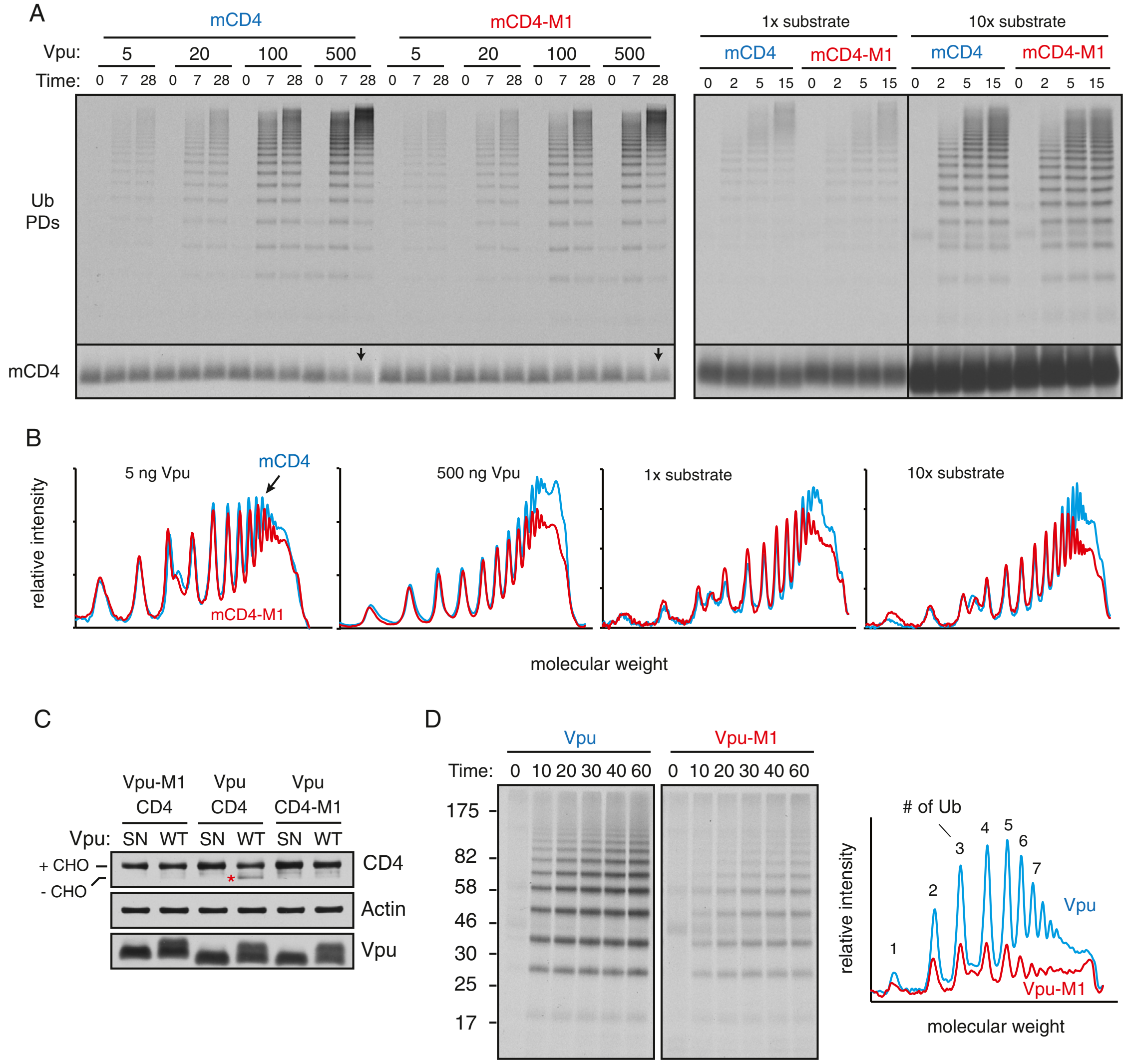

Figure S5. Comparison of Substrate Discrimination in Different Experimental Systems, Related to Figure 4

(A) In the left panel, fixed amounts of radiolabeled mCD4 or mCD4-M1 were coreconstituted with varying amounts of Vpu (between 5 to 500 ng) into proteoliposomes and analyzed for ubiquitination as in Figure 4A. The right panel contains a fixed amount of Vpu (20 ng) and two different amounts of the substrate (either mCD4 or mCD4-M1). The ubiquitin pulldowns (Ub PDs) are shown in the top panels, and the unmodified substrate is shown in the bottom panels. Note that ubiquitination efficiency increases with increasing Vpu concentrations, while the overall signal (but not efficiency) is increased with increasing substrate concentrations. At the highest Vpu concentration, ubiquitination efficiency is greater than 50%, being visible as a clear decrease in the unmodified substrate (arrowhead). The reason for lower substrate turnover in the lower Vpu concentrations is because Vpu is constrained to liposomes, meaning that a Vpu molecule in one liposome cannot access potential substrates in other liposomes. Conversely, mCD4 in a liposome without Vpu (as is more likely at low Vpu concentrations) cannot be ubiquitinated.

(B) Densitometry profiles of selected reactions from A illustrating that very little difference is observed between mCD4 and mCD4-M1 regardless of the ratio of substrate to Vpu. The only systematic difference is seen with long ubiquitin chains, consistent with slightly lower processivity of ubiquitin addition for mCD4-M1 relative to mCD4.

(C) The indicated CD4 and Vpu construct pairs were coexpressed, subjected to proteasome inhibition for 14 hr with 20 μM MG132, and analyzed by immunoblotting. The deglycosylated CD4 band (red asterisk), seen only with WT Vpu, is indicative of dislocation from the ER.

(D) mCD4 was in vitro translated into microsomes isolated from cells expressing Vpu or Vpu-M1 and subjected to ubiquitination reactions. The profiles from the 20 min time point are shown in the densitometry traces on the right.

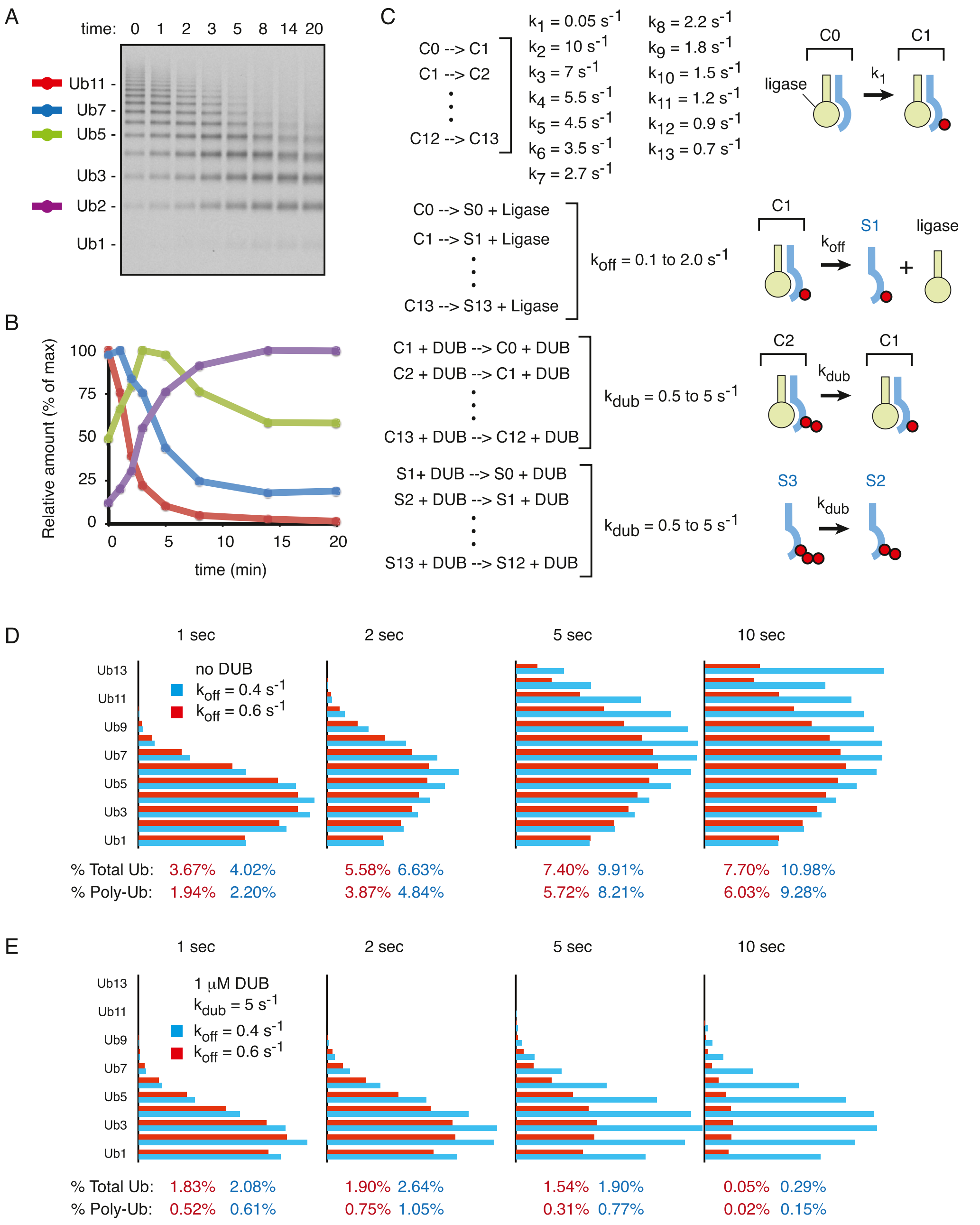

Figure S6. Experimental and In Silico Analysis of DUB Activity, Related to Figure 5

(A) Deubiquitination of mCD4 by HEK293 cytosol. Proteoliposomes containing ubiquitinated mCD4 generated as in Figure S4B were isolated and incubated with HEK293 cytosol. Aliquots at different time points were analyzed by ubiquitin pulldowns and autoradiography.

(B) The Ub2 (i.e., substrate containing two ubiquitins), Ub5, Ub7, and Ub11 bands from A were quantified, normalized to maximal intensity of that species, and plotted. This analysis indicates that ubiquitins are successively removed from the distal ends of the polyubiquitin chains.

(C) The reactions used to model the ubiquitination and deubiquitination reactions illustrated in Figure 5B. In this model, C0 to C13 represent substrate-ligase complexes containing between 0 to 13 ubiquitins on the substrate. Hence, the reaction 'C0 = C1' represents a single ubiquitin addition. The kcat values for these reactions are indicated. The reactions of the type 'C0 = S0 + Ligase' are dissociation reactions of ligase from substrate (i.e., S0 to S13, depending on the number of ubiquitins attached). The deubiquitination reactions are allowed to occur on substrates that are either free or in a complex with ligase.

(D and E) Analysis of the normalized ubiquitin profiles generated by the model at different time points for the indicated parameters without or with DUB activity. The absolute amount of total and poly-ubiquitinated species at each time point is indicated below the respective graphs. Note that in the absence of DUBs (D), ubiquitination is very similar for the two substrates of differing K_{off} values. Only a modest difference is observed at later time points. By contrast, high DUB activity (E) results in a substantial (~7-8 fold) difference in polyubiquitination over time. However, this comes at the cost of a marked loss in overall ubiquitination efficiency.

Cell

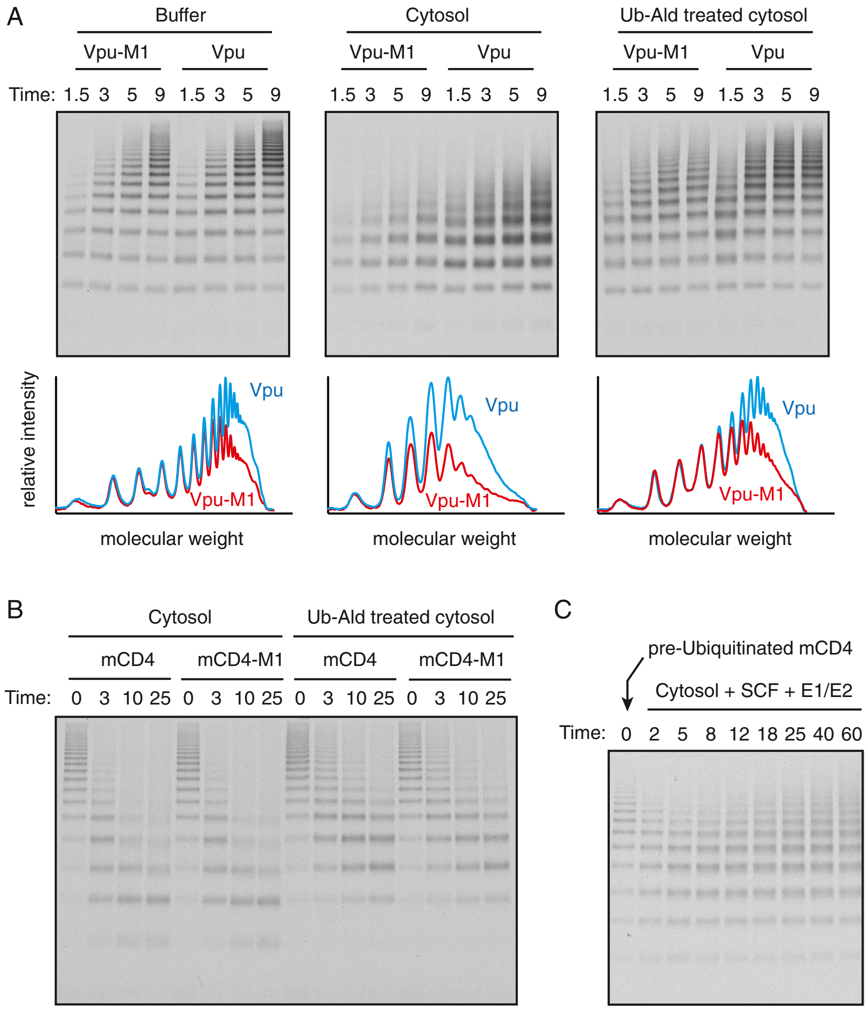

Figure S7. DUBs Enhance Substrate Discrimination In Vitro, Related to Figure 6

(A) Radiolabeled mCD4 isolated from in vitro translation reactions was coreconstituted with recombinant Vpu or Vpu-M1 into liposomes and subjected to ubiquitination in the presence of BSA, HEK293 cell cytosol, or cytosol pretreated with ubiquitin-aldehyde. The reaction at different time points was stopped and the analyzed for ubiquitinated mCD4 via pulldowns of His6-tagged ubiquitin (Ub PDs). Total mCD4 in the reaction is shown in the bottom panel. The graphs below the autoradiographs depicts the densitometry profiles of the 5 min samples.

(B) Proteoliposomes containing ubiquitinated mCD4 or mCD4-M1 generated as in Figure S4B were isolated and incubated with HEK293 cytosol, or cytosol pretreated with ubiquitin-aldehyde. Samples were removed at different time points and analyzed for ubiquitinated products by pulldowns and autoradiography. Note that deubiquitination activity is reduced, but not completely eliminated, by ubiquitin-aldehyde pretreatment.

(C) An experiment as in B except that the cytosol was supplemented with the SCF complex and E1/E2 enzymes. Note that initially, deubiquitination is observed, but over time, this activity is counteracted by the SCF.
